# SARS-CoV-2 S protein activates NLRP3 inflammasome and deregulates coagulation factors in endothelial and immune cells

**DOI:** 10.1186/s12964-023-01397-6

**Published:** 2024-01-15

**Authors:** Alicia Villacampa, Enrique Alfaro, Cristina Morales, Elena Díaz-García, Cristina López-Fernández, José Luis Bartha, Francisco López-Sánchez, Óscar Lorenzo, Salvador Moncada, Carlos F. Sánchez-Ferrer, Francisco García-Río, Carolina Cubillos-Zapata, Concepción Peiró

**Affiliations:** 1 Department of Pharmacology, School of Medicine, Universidad Autónoma de Madrid, Madrid, Spain; 2 Respiratory Diseases Group, Respiratory Service, La Paz University Hospital, IdiPAZ, Madrid, Spain; 3 Biomedical Research Networking Center on Respiratory Diseases (CIBERES), Madrid, Spain; 4 Department of Obstetrics and Gynecology, School of Medicine, Universidad Autónoma de Madrid, Madrid, Spain; 5 Gynecology and Obstetrics Service, La Paz University Hospital, Madrid, Spain; 6 Laboratory of Diabetes and Vascular pathology, IIS-Fundación Jiménez Díaz, Madrid, Spain; 7 Biomedical Research Networking Centre on Diabetes and Associated Metabolic Disorders (CIBERDEM), Madrid, Spain; 8 Department of Medicine, School of Medicine, Universidad Autónoma de Madrid, Madrid, Spain; 9 Vascular Pharmacology and Metabolism (FARMAVASM) group, IdiPAZ, Madrid, Spain

**Keywords:** Blood coagulation factors, SARS-CoV-2, Endothelial cells, NLRP3 inflammasome, Monocytes

## Abstract

**Background:**

Hyperinflammation, hypercoagulation and endothelial injury are major findings in acute and post-COVID-19. The SARS-CoV-2 S protein has been detected as an isolated element in human tissues reservoirs and is the main product of mRNA COVID-19 vaccines. We investigated whether the S protein alone triggers pro-inflammatory and pro-coagulant responses in primary cultures of two cell types deeply affected by SARS-CoV-2, such are monocytes and endothelial cells.

**Methods:**

In human umbilical vein endothelial cells (HUVEC) and monocytes, the components of NF-κB and the NLRP3 inflammasome system, as well as coagulation regulators, were assessed by qRT-PCR, Western blot, flow cytometry, or indirect immunofluorescence.

**Results:**

S protein activated NF-κB, promoted pro-inflammatory cytokines release, and triggered the priming and activation of the NLRP3 inflammasome system resulting in mature IL-1β formation in both cell types. This was paralleled by enhanced production of coagulation factors such as von Willebrand factor (vWF), factor VIII or tissue factor, that was mediated, at least in part, by IL-1β. Additionally, S protein failed to enhance ADAMTS-13 levels to counteract the pro-coagulant activity of vWF multimers. Monocytes and HUVEC barely expressed angiotensin-converting enzyme-2. Pharmacological approaches and gene silencing showed that TLR4 receptors mediated the effects of S protein in monocytes, but not in HUVEC.

**Conclusion:**

S protein behaves both as a pro-inflammatory and pro-coagulant stimulus in human monocytes and endothelial cells. Interfering with the receptors or signaling pathways evoked by the S protein may help preventing immune and vascular complications driven by such an isolated viral element.

Video Abstract

**Supplementary Information:**

The online version contains supplementary material available at 10.1186/s12964-023-01397-6.

## Background

Corona Virus Disease 2019 (COVID-19), was reported from Wuhan city, China, and has caused over 750 million confirmed infections and nearly 7 million deaths worldwide according to the World Health Organization Coronavirus (COVID-19) Dashboard [1]. The enormous impact of COVID-19 and its sequalae on human health and the socioeconomic system [2] has raised massive interest in better understanding the pathophysiological mechanisms of the disease.

Severe acute respiratory syndrome coronavirus 2 (SARS-CoV-2), the causative agent of the COVID-19 pandemia, is a member of the coronavirus family. As such it is covered by a crown of spike (S) protein, which is composed of two main domains, S2 and S1 [3, 4]. The latter contains the receptor binding domain (RBD) that forms a trimer and attaches to the host cell receptor, while the S2 domain mediates viral cell membrane fusion and entry [3, 4]. Angiotensin converting enzyme 2 (ACE2) was initially identified as a major receptor allowing for SARS-CoV-2 entry in different human cell types [5, 6]. However, other cell surface molecules have been acknowledged to mediate the recognition between the viral S protein and host cells, including toll-like receptor 4 (TLR4), basigin or cluster of differentiation (CD) 147, and dipeptydilpeptidase-4 or CD26 [7, 8]. Also, the concept has emerged that specific viral elements alone, including the S protein, might interact with host cell receptors to trigger intracellular responses [9].

Together with the description of respiratory symptoms and acute respiratory distress syndrome (ARDS), very early since the beginning of the COVID-19 pandemia, endothelial injury was a primary finding in patients infected by SARS-CoV-2 [10]. Postmortem histology revealed viral inclusions in endothelial apoptotic cells, microvascular lymphocytic endotheliitis, and the infiltration of inflammatory immune cells around the vessels and the endothelial layer together with microthrombi formation [11, 12]. Since then, a large series of clinical observations have identified the vasculature as one of the main trans-organ systems affected by SARS-CoV2 infection as well as a major cause of sequalae following COVID-19 [13, 14].

Inflammatory responses in severe COVID-19 patients are characterized by intense immune cell recruitment and enhanced levels of inflammatory markers including C-reactive protein, ferritin and cytokines, associated with hyper-coagulation state [15, 16]. Elevated circulating levels of pro-coagulant factors such as von Willebrand factor (vWF), factor VIII (FVIII) or tissue factor (TF) were found in a high number of patients with COVID-19 [17, 18]. While FVIII and vWF are mainly produced by endothelial cells, TF can also be released by other activated cell types, particularly activated immune cells [19]. The coordinated study of inflammatory and coagulant factors that trigger endothelial hyper-activation and thrombosis seems nowadays essential to understand the complex pathophysiology of COVID-19 and its sequalae and to design more rationale and better targeted therapeutical treatments.

The NLR family pyrin domain-containing 3 (NLRP3) inflammasome system, a first-line sensor of the innate immune response, is currently considered as a key driver of vascular inflammation and endothelial dysfunction [20] and a relevant player in multiple pathologies including atherosclerosis and other cardiovascular diseases [21], diabetes mellitus [22], obstructive sleep apnea [23], or viral infections including COVID-19 [24]. After a first priming phase to enhance some cellular components of the system, such as NLRP3 or the inactive precursor of interleukin-1β (pro-IL-1β), the inflammasome need to assemble into a functional multi-protein structure involving NLRP3 and other proteins, including adaptor molecule apoptosis-associated speck-like protein (ASC). This, in turn, leads to the activation of caspase-1, which cleaves pro-IL-1β and pro-IL-18 into their mature active forms [25] and, in monocytes/macrophages, triggers gasdermin D activation, favoring the formation of pores in the cell membrane that permit the release of pro-inflammatory cytokines [26, 27]. In the context of COVID-19 disease, activation of NLRP3 inflammasome can be triggered by the massive liberation of proinflammatory cytokines [28]. However, the role of other activators, such as the viral S protein, which has been detected in the circulation of infected patients or in tissues after SARS-CoV-2 infection [29] and is the main product of mRNA vaccines against COVID-19, remains elusive.

The aim of the present study was to address whether the SARS-CoV2 S protein can, as an isolated viral element, directly activate pro-inflammatory and pro-thrombotic signaling in primary human endothelial and immune cell cultures, with special attention to the role of the NLRP3 inflammasome activation and the release of pro-coagulant factors.

## Material and methods

*(for more details, please see* *Supplemental Material**)*

### Human umbilical vein endothelial cells culture

Human umbilical vein endothelial cells (HUVEC) were isolated from umbilical cords from donors at Hospital Universitario La Paz (Spain, Madrid) with informed consent, following the Spanish legislation and under approval of the appropriate Research Ethics Committee as previously described [30].

### PBMCs, monocytes isolation and cell cultures

Peripheral blood mononuclear cells (PBMC) and monocyte isolation and culture were obtained by venipuncture from peripheral vein from healthy subjects (aged 18–65) as described in Supplemental Methods.

### Western blot

HUVEC or enriched monocytes were lysed and protein lysates were separated and quantified by Western blot [31], as described in Supplemental Methods.

### Visualization of NLRP3 activation by indirect immunofluorescence

Once NLRP3 inflammasome activation is triggered, the ASC protein assembles, and forms toroidal structures known as specks [32] that were visualized in HUVEC by indirect immunofluorescence, as previously described [33].

### TLR4 and NLRP3 inhibition assays

The pharmacological inhibition of TLR4 and NLRP3 inflammasome by means of TAK242 and MCC950, respectively, and TLR4 silencing were performed as described in Supplemental Methods.

### Statistical analysis

Variables were analyzed for normality using Shapiro-Wilks’ test. For variables presenting normality, mean differences were evaluated using paired t-test, for pairs comparisons, and using repeated measures ANOVA (R-M-ANOVA) with Tuckey’s test multiple comparison, for more than two groups comparison. For variables not presenting normality, the Wilcoxon’s test was used to assess differences between two groups. In line, the Friedman’s test was used to analyze differences among more than two groups, including the Dunn’s test for multiple comparisons.

## Results

### S protein promotes the priming of the NLRP3 inflammasome in HUVEC

**Fig. 1:**
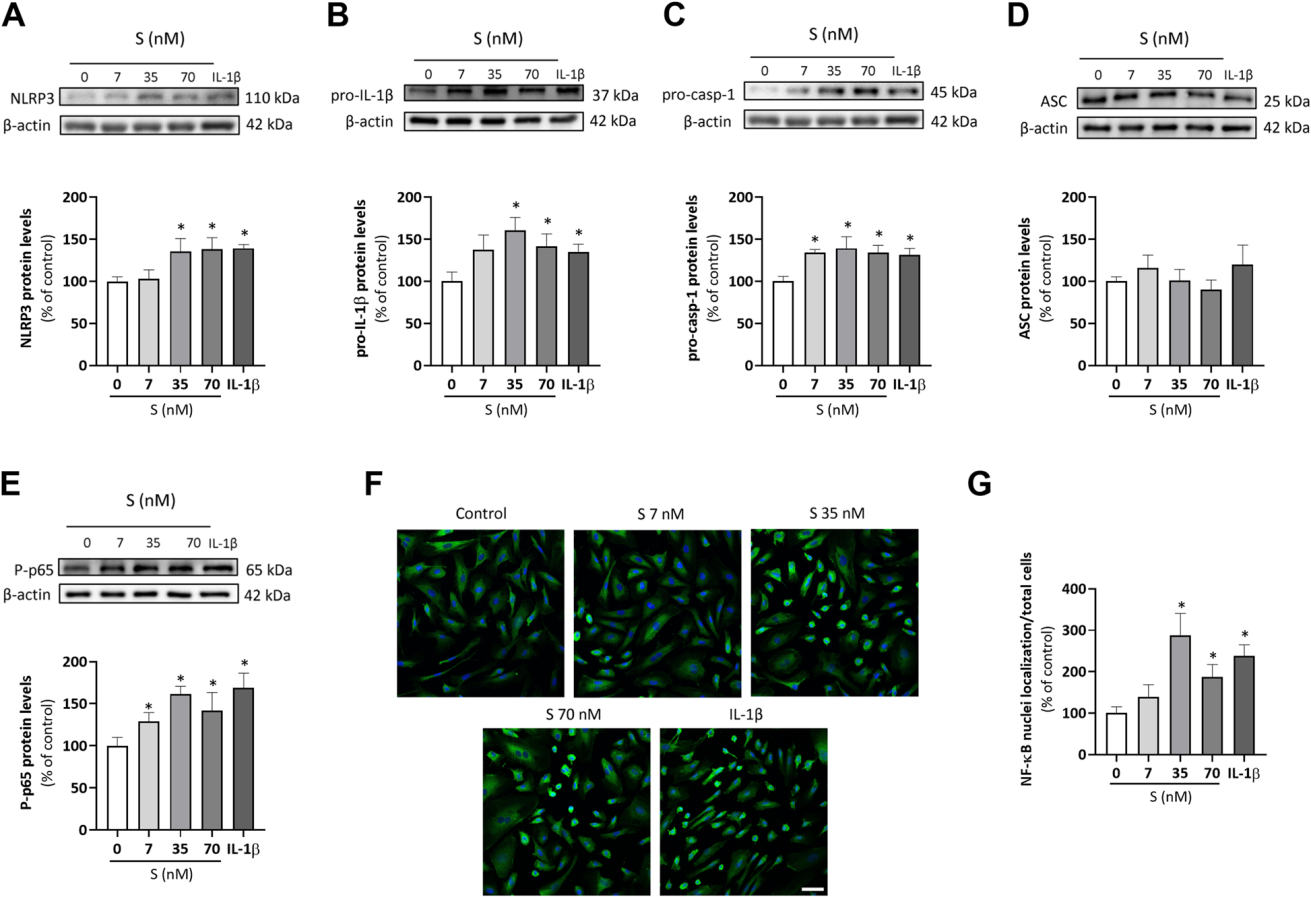
S protein promotes NLRP3 priming and NF-κB activation in primary HUVEC cultures. Human umbilical vein endothelial cells (HUVEC) were treated with viral S protein at 7, 35 and 70 nM and IL-1β at 2.5 ng/mL for 18 h and the protein levels of the NLRP3 inflammasome system components **A** NLRP3 (*n* = 5), **B** pro-IL-1β (*n* = 4), **C** pro-casp-1 (*n* = 5), and **D** ASC protein levels (*n* = 3) were analyzed in total cell lysates by Western blot. In addition, NF-κB activation was quantified by means of **E** phospho-p65 (P-p65) protein levels by Western blot (*n* = 3) and **F** visualized by indirect immunofluorescence as the translocation of p65 (green) to cell nuclei countersatained with DAPI (blue). Representative images from confocal microscopy of p65 immunofluorescence staining are shown. Scale bar represents 50 μm (**G**). For quantification of cells with nuclear localization of p65 at least 200 cells per treatment were counted (*n* = 4). For Western blots, representative gels are shown on top of the corresponding graphs, with β-actin used as a loading control. Bar graphs represent mean ± SEM. Statistical differences were analyzed by t-test. *p < 0.05 versus unstimulated control

In HUVEC exposed to S protein (7, 35 and 70 nM) a concentration-dependent increase in the protein levels of both NLRP3 and pro-IL-1β was observed from a threshold concentration of 35 nM (Fig. 1A and B). The expression of pro-caspase-1 was equally enhanced by S protein (Fig. 1C), while ASC levels remained unchanged (Fig. 1D). The pro-inflammatory cytokine IL-1β (2.5 ng/mL) elicited similar effects on the priming of the different NLRP3 inflammasome components (Figs. 1A to D).

### S protein activates NF-κB in HUVEC

Since nuclear factor-kappa B (NF-κB) is a major transcription factor in inflammatory responses that can regulate the expression of several NLRP3 inflammasome components [28], we next assessed the capacity of SARS-CoV2 S protein to activate the NF-κB pathway. Fig. 1E shows how S protein increased in a concentration-dependent manner the levels of phosphorylated-p65 (pp65) used as a marker of NF-κB activation. The translocation of active pp65 from the cytoplasm to the nucleus was visualized by indirect immunofluorescence (Fig. 1F) and quantified by image analysis (Fig. 1G). IL-1β (2.5 ng/mL) was used as a positive control of NF-κB activation (Figs. 1E to G).

### S protein promotes the activation of the NLRP3 inflammasome in HUVEC

**Fig. 2:**
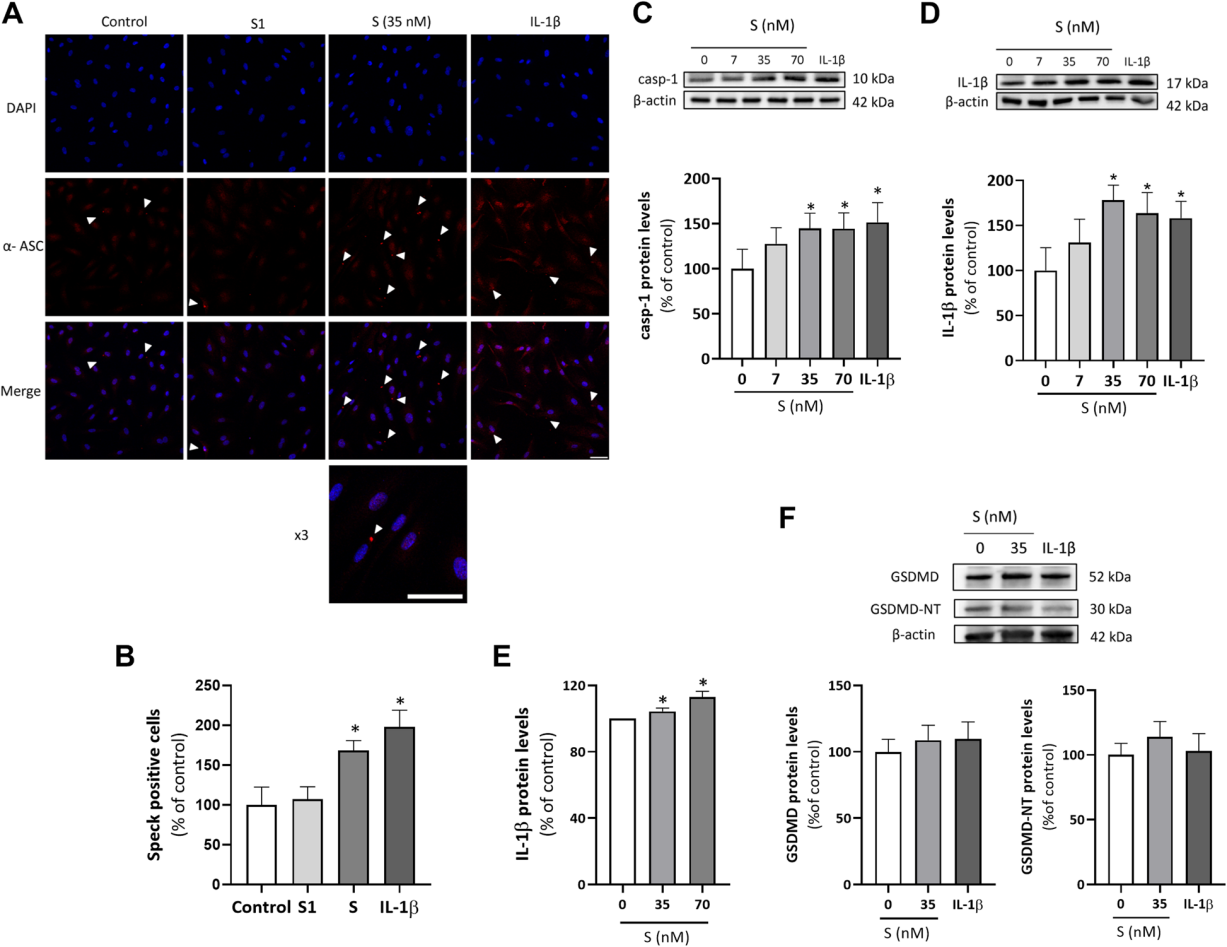
S protein promotes the activation of the NLRP3 inflammasome in HUVEC. Human umbilical vein endothelial cells (HUVEC) were treated with viral S protein (35 nM), IL-1β (2.5 ng/mL) or the S1 fragment of S protein (35 nM) for 18–24 h. **A** The formation of toroidal specks corresponding to activated NLRP3 inflammasome were visualized by indirect immunofluorescence against ASC (red) using a confocal microscope. Nuclei were counter-stained with DAPI (blue). Scale bar represents 50 μm **B** NLRP3 inflammasome activation was quantified by manual blind scoring of 27 radial distributed fields per sample as the number of ASC speck-positive cells. White arrows indicate speck positive cells. Scale bar represents 50 μm. In addition, the cellular protein levels of **C** active cleaved caspase-1 (casp-1) (*n* = 6) and **D** mature IL-1β (*n* = 4). **E** IL-1β released to the cell supernatants was quantified by ELISA (*n* = 5–8). **F** Gasdermin D (GSDMD) and cleaved GSDMD-NT (*n* = 8) were determined by Western blot in total cell lysates from HUVEC treated with 7, 35 and 70 nM S protein or 2,5 ng/mL IL-1β. Representative gels are shown on top of the corresponding graphs, with β-actin used as a loading control. Bar graphs represent mean ± SEM. Statistical differences were analyzed by t-test. *p < 0.05 versus control with no S protein

Upon activation, NLRP3 protein oligomerizes and interacts with ASC to assemble into a multiprotein scaffold (ASC speck) wherein caspase-1 is activated to process pro-IL-1β and pro-IL-18 into their mature forms [32]. Indeed, S protein (35 nM) promoted the formation of such toroidal-shaped specks characteristic of assembled and functional NLRP3 inflammasome as visualized by ASC protein immunostaining (Fig. 2A) and its subsequent quantification (Fig. 2B). In accordance, higher levels of active caspase-1 and mature IL-1β were also found in endothelial cells stimulated with increasing concentrations of the viral S protein (Fig. 2C and D, respectively). IL-1β levels were found enhanced in cell supernatants (Fig. 2E), while no significant changes were observed in the cellular content of the protein gasdermin D (GSDMD) or its pore-forming N-terminal cleavage product (GSDMD-NT) after S protein or stimulation IL-1β (Fig. 2F).

We next studied whether the S1 fragment of the S protein, which contains the RBD that binds the host cell receptors, was sufficient to trigger NLRP3 inflammasome activation in HUVEC. The S1 fragment did not induce the formation of ASC-specks by itself (Fig. 2A and B), suggesting that the whole S protein or at least a trimeric S1 conformation may be required to exert such an activation of the NLRP3 inflammasome complex.

### S protein enhances the levels of coagulation factors and reduces ADAMST-13 availability in HUVEC

**Fig. 3:**
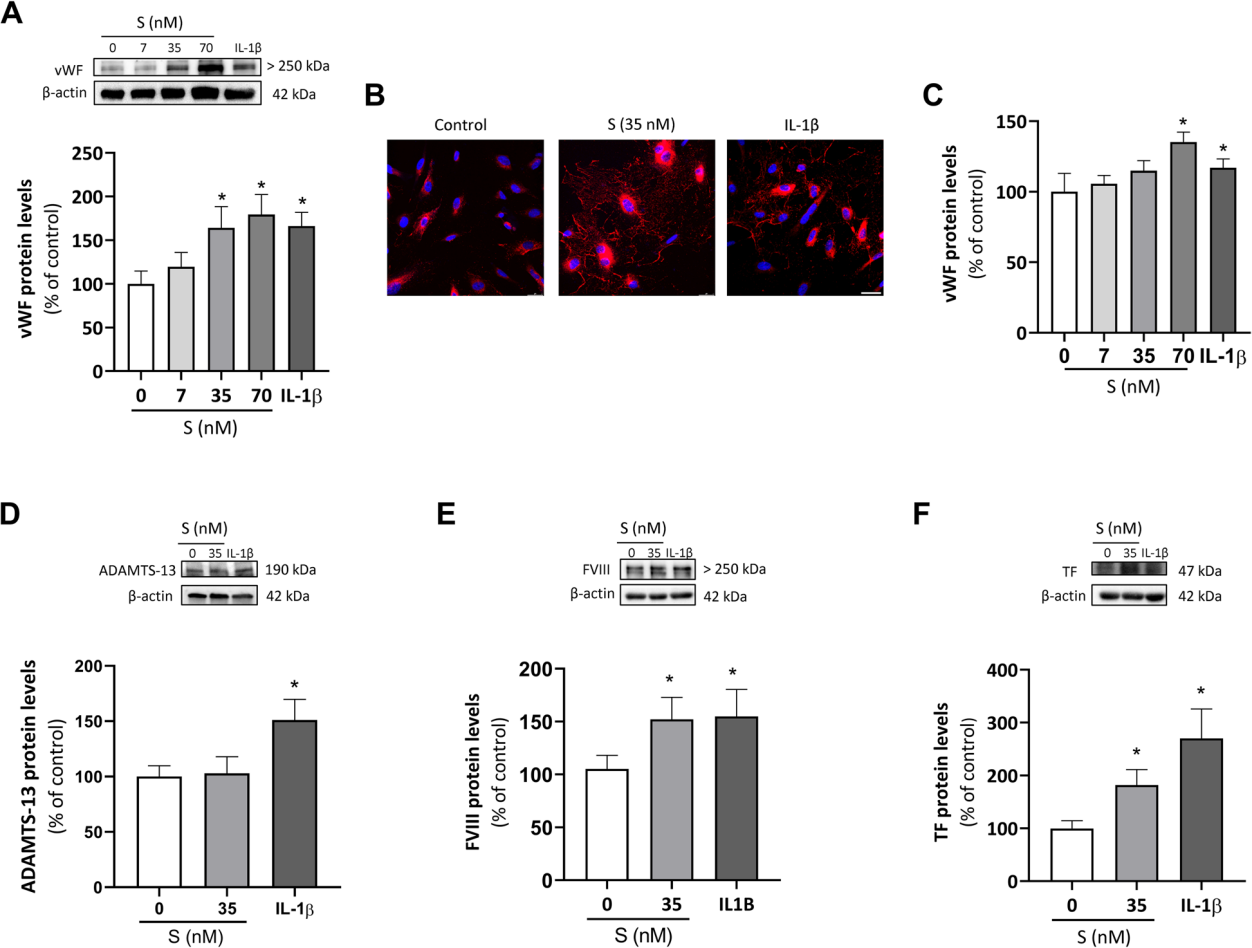
The activation NLRP3 inflammasome and the release of IL-1β increase coagulation factors mediated by S protein. Human umbilical vein endothelial cells (HUVEC) were treated with viral S protein at 7, 35 and 70 nM or IL-1β at 2.5 ng/mL for 18 h. The levels of von Willebrand Factor (vWF) were determined **A** by Western blot in total cell lysates (*n* = 6), and **B** visualized in HUVEC stimulated with S (35 nM) or IL-1β (2.5 ng/mL) by indirect immunoflorescence (red). Representative images are shown in which vWF can be seen both as granules within the HUVEC cytoplasm and multimeric filaments in the extracellular space. Nuclei were counter-stained with DAPI (blue). Scale bar represents 50 μm. **C** vWF was also measured by ELISA in cell supernatants (*n* = 4). In the same experimental conditions, the protein levels of **D** ADAMTS-13, **E** FVIII, and **F** TF were quantified in total cell lysates by Western blot. Representative gels are shown on top of the corresponding graphs, with β-actin used as a loading control. Bar graphs represent mean ± SEM. Statistical differences were analyzed by t-test. *p < 0.05

Endothelial cells can synthesize and release a number of key pro-coagulant proteins that can participate in prothrombotic events. HUVEC challenged with the S protein exhibited a concentration-dependent increase in the vWF content as determined by Western blot (Fig. 3A). Moreover, as visualized by immunofluorescence, vWF was detected in intracellular granules but also in the extracellular space forming multimeric filaments, which were mainly visible in cultures stimulated with the S protein (Fig. 3B). The increased secretion of vWF to the cell supernatants induced by the viral protein was confirmed and quantified by ELISA (Fig. 3C). IL-1β exerted similar effects on vWF levels and secretion, although with a less intense extracellular vWF filament staining (Figs. 3A to C). Additionally, IL-1β enhanced the endothelial content in A disintegrin and metalloprotease with a thrombospondin type 1 motif, member 13 (ADAMST-13), a primary molecular regulator that attenuates vWF activity by cleaving multimers [34], while this effect was not apparent in HUVEC exposed to S protein (Fig. 3D). In addition, S protein enhanced the content of FVIII and TF, an initiator of the extrinsic coagulation pathway, in HUVEC, as also did IL-1β (Fig. 3E and F).

Because of the connection reported between inflammation and coagulation [35], we next addressed whether activation of the NLRP3 inflammasome pathway could be at the basis of the enhanced production of pro-coagulant factors induced by S protein. In the presence of the NLRP3 inflammasome inhibitor MCC950 (1 μM) a trend was observed towards the reduction of vWF (by 22.86%) and TF (by 44.34%) levels induced by S protein, although statistical significancy was not reached (Fig. S1A and S1B). However, the IL-1R blocker anakinra (1 μg/mL) significantly prevented the effect of the viral protein on TF levels and reduced vWF by 30.79% (Fig. S1B). As expected, anakinra blunted the stimulatory action of IL-1β thus confirming the capacity of the drug to block IL-1R receptors (Fig. S1A and S1B).

### S protein activates NF-κB pathway and triggers NLRP3 inflammasome activation and TF release in human monocytes

**Fig. 4:**
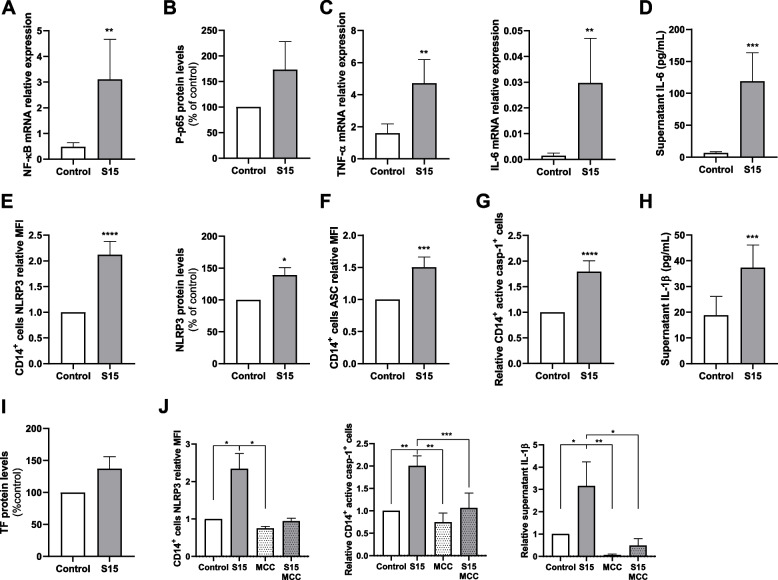
NF-κB pathway and NLRP3 inflammasome components in S protein stimulated monocytes. Enriched monocytes from healthy donor were cultured under control conditions or stimulated with S protein 15 nM (S15). **A** NF-κB mRNA expression analysis by qPCR in enriched monocytes cultured for 16 h (*n* = 12). **B** Western blot protein values of phosphorylated NF-κB p65 particle (pp65) relative to β-actin protein values from lysates of enriched monocytes cultured for 16 hours (*n* = 3). **C** mRNA expression analysis by qPCR of TNF-α (*n* = 12) and IL-6 (*n* = 9) in enriched monocytes cultured for 3 hours. **D** IL-6 concentration in supernatant from enriched monocytes cultured for 16 hours (*n* = 12). **E** Left: CD14^+^ gated-cells normalized mean fluorescent intensity (MFI) of NLRP3 (*n* = 20); Right: normalized NLRP3 to β-actin protein ratio determined by Western blot from enriched monocyte’s lysates cultured for 16 hours (*n* = 7). **F** CD14^+^ gated cells normalized mean fluorescent intensity (MFI) of ASC (*n* = 12). **G** Normalized amount of active caspase positive (Casp-1^+^) CD14^+^ cells analyzed by flow cytometry from enriched monocytes cultured for 16 hours (*n* = 20). **H** Supernatant IL-1β concentration measured by CBA (*n* = 12). **I** Normalized TF to β-actin protein ratio determined by Western blot from enriched monocytes lysates cultured for 16 hours (*n* = 5). Differences were analyzed using Wilcoxon’s paired test. **J** Enriched monocytes were stimulated with S protein or control, treated or not with MCC950 and cultured for 16 h. Left: CD14^+^ gated-cells normalized mean fluorescent intensity (MFI) of NLRP3 (*n* = 12); center: normalized amount of Casp-1^+^ CD14^+^ cells analyzed by flow cytometry (*n* = 15); right: supernatant IL-1β concentration measured by CBA (*n* = 6). Differences were analyzed by repeated measures ANOVA and Tukey’s multiple comparison test. All data are represented as mean ± Standard Error of the Mean (SEM). Only statistically significant differences are stated: *p < 0.05, **p < 0.01., ***p < 0.001 and ****p < 0.0001

We next studied the effects of the S protein in monocytes as key components of the innate immune response triggering pro-inflammatory pathways. We first performed an in vitro model using enriched human monocytes to determine the kinetic time-course of NF-κB expression (Fig. S2A) and found elevated NF-κB mRNA expression after cells were exposed to S protein (15 nM) for 16 h (Fig. 4A). The activation of the NF-κB pathway was confirmed by the increased pp65 levels (Fig. 4B and Fig. S2B). Active NF-κB is able to translocate to the nucleus where it triggers the transcription of several response genes including the tissue necrosis factor alpha (TNF-α) and IL-6 inflammatory cytokines. Accordingly, we observed increased TNF-α and IL-6 mRNA (Fig. 4C and Figs. S2C and S2D), together with higher protein levels of IL-6 in supernatants of monocytes treated with S protein (Fig. 4D). Altogether S protein triggered NF-κB pathway in human monocytes leading to inflammatory cytokines production.

NF-κB pathway is part of the NLRP3 inflammasome priming. In accordance with the results in HUVEC, we observed an increased NLRP3 expression in monocytes stimulated with S protein, as measured by flow cytometry and Western blot (Fig. 4E, Fig. S3A and Fig. S4A), together with an over-expression of ASC (Fig. 4F and Fig. S3B). Additionally, increased mRNA expression of inflammasome components (NLRP3, ASC, caspase-1) and TF was observed after S protein stimulation (Fig. S4B).

To assess the ability of S protein to also trigger NLRP3 inflammasome activation in human monocytes, we performed a FAM-FLICA caspase-1 assay which uses fluorescent inhibitor probe FAM-YVAD-FMK, capable of specifically labeling active caspase-1. By flow cytometry, we observed an elevated number of monocytes positively stained for active caspase-1 after stimulation by S protein (Fig. 4G and Fig. S3C). In accordance, IL- 1β levels were enhanced in the supernatants from monocytes treated with S protein as compared with untreated controls (Fig. 4H). Moreover, treated monocytes presented increased levels of TF, measured by Western blot (Fig. 4I and Fig. S4C). Finally, to assess the role of NLRP3 inflammasome in the S protein-triggered increase of active caspase-1 and supernatant IL-1β, we performed an experiment including the specific NLRP3 inhibitor MCC950, which was capable to reduce NLRP3 expression and to limit active caspase-1 production and IL-1β release by monocytes stimulated with S protein (Fig. 4J). Moreover, the use of caspase-1 inhibitor, Ac-YVAD-cmk, reduced the cellular content of caspase-1 as well as the amount of IL-1β in cell supernatants, minimizing S protein effect (Fig. S4D). Altogether the data point out the ability of S protein to trigger the priming and activation of NLRP3 inflammasome in human monocytes, resulting in the release of active inflammatory cytokines and the production of TF, which is involved in the coagulation cascade.

### TLR4 receptors mediate the pro-inflammatory and pro-coagulant action of S protein in monocytes but not in HUVEC

We next aimed to identify in monocytes and HUVEC potential cell receptors capable to interact with S protein to trigger NLRP3 inflammasome inflammation and the release of pro-coagulant factors. ACE2 has been proposed as one of the main human host cell receptors for SARS-CoV2 [5, 6]. However, its expression was not detectable in both HUVEC and PBMC primary cultures as assessed by real time-quantitative PCR (RT-qPCR) (Fig. S5).

**Fig. 5:**
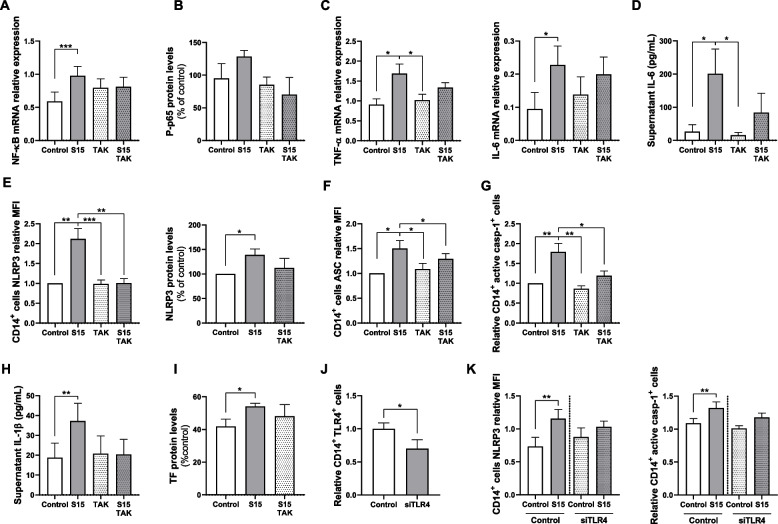
Effect of S protein stimulation over monocytes treated with TLR4 inhibitor TAK242. (A-I) Enriched monocytes from healthy donor were cultured for 16 h and were stimulated or not with S protein (S15) and treated or not with TLR4 inhibitor (TAK242). **A** NF-κB mRNA expression analysis by qPCR. (*n* = 9). **B** Western blot protein values of phosphorylated NF-κB p65 particle (pp65) relative to β-actin protein values (*n* = 3). **C** mRNA expression analysis by qPCR of TNF-α (*n* = 10) and IL-6 (*n* = 7). **D** IL-6 concentration in supernatant from enriched monocytes (*n* = 10). **E** Left: CD14^+^ gated-cells normalized mean fluorescent intensity (MFI) of NLRP3 (*n* = 20); right: normalized NLRP3 to β-actin protein ratio determined by Western blot (*n* = 7). **F** CD14^+^ gated cells normalized mean fluorescent intensity (MFI) of ASC (*n* = 12). **G** Normalized amount of active caspase positive (Casp-1^+^) CD14^+^ cells analyzed by flow cytometry (=20). **H** Supernatant IL-1β concentration measured by CBA (*n* = 12). **I** Normalized TF to β-actin protein ratio determined by Western blot (*n* = 5). Differences were analyzed by repeated measures ANOVA and Tukey’s multiple comparison test. **J-K** Enriched monocytes from healthy donor were transfected in absence (control) or presence of TLR4 siRNA (siTLR4) and cultured for 16 h with S15 or not. **J** Normalized amount of CD14^+^ cells expressing surface TLR4 (*n* = 7). **K** Left: CD14^+^ gated-cells normalized mean fluorescent intensity (MFI) of NLRP3 (*n* = 7): right: normalized amount of active casp-1^+^ CD14^+^ cells analyzed by flow cytometry (*n* = 7). Differences were analyzed using Wilcoxon’s paired test. All data are represented as mean ± SEM. Only statistically significant differences are stated: *p < 0.05, **p < 0.01, and ***p < 0.001

Based on the capacity of S protein to prime and activate the innate immune system complex NLRP3 inflammasome, we next tested the implication of TLR4, a major receptor of this system abundantly expressed in monocytes and also present in cardiac and vascular cells [36]. In monocytes, resatorvid (TAK242; 5 μM), a selective TLR4 inhibitor, significantly reduced the upregulation of NF-κB mRNA (Fig. 5A) and the enhanced NF-κB p65 phosphorylation (Fig. 5B) induced by the S protein. Moreover, the drug prevented mRNA overexpression of TNF-α and IL-6 (Fig. 5C) and reduced the concentration of IL-6 in supernatants stimulated by S protein (Fig. 5D).

Since TLR4 inhibition limited the activation of NF-κB, we next analyzed the impact of the drug on NLRP3 priming and activation. TAK242 abrogated the ability of S protein to overexpress NLRP3 at both protein (Fig. 5E and Fig. S4A) and mRNA expression level (Fig. S6A) and limited ASC expression induced by the viral protein (Fig. 5F and Fig. S6A). Accordingly, S protein was unable to trigger the accumulation of active caspase-1 (Fig. 5G) or the overexpression of caspase-1 mRNA (Fig. S6A) in the presence of TAK242. Lastly, TLR4 inhibition reduced the concentration of IL-1β in supernatants from monocytes stimulated with S protein (Fig. 5H), as well as TF protein levels (Fig. 5I and Fig. S4C) and TF mRNA expression induced by the viral protein (Fig. S6A). Altogether, TAK242 impaired the ability of S protein to activate NF-κB pathway and trigger NLRP3 inflammasome priming and activation, pointing out the importance of TLR4 receptor in the interaction of S protein with monocytes.

In order to confirm the observations obtained with the TLR4 pharmacological inhibition we next used a molecular approach, where we blocked TLR4 expression in monocytes using specific TLR4 short interfering RNA (siTLR4). After confirming by flow cytometry that TLR4 was indeed reduced in monocytes surface after transfection with siTLR4 (Fig. 5J), we observed that NLRP3 levels induced by S protein were limited in monocytes transfected with siTLR4 (Fig. 5K). Similarly, active caspase-1 accumulation caused by S protein was abrogated in siTLR4 monocytes (Fig. 5K). We also analyzed, by RT-qPCR, the effect of siTLR4 transfection in the mRNA expression of NLRP3 components. In accordance with the previous results, mRNA expression of NLRP3, ASC, caspase-1 and TF was reduced in siTLR4 monocytes stimulated with S protein compared to control monocytes equally stimulated (Fig. S6B).

Unlike to that observed in monocytes, the pharmacological TLR4 blockade with TAK242 in HUVEC primary cultures did not result in restricted priming or activation of the NLRP3 inflammasome (Figs. S7A and S7B) nor did it reduce the induction of vWF elicited by the S protein after (Fig. S7C). These results did not point at a major role for TLR4 in HUVEC, thus indicating the existence of other potential receptors for the S protein in this cell type.

## Discussion

In this study we demonstrate that SARS-CoV-2 S protein can act as an isolated element that stimulates per se pro-inflammatory and pro-coagulant responses in human primary cultures of monocytes and endothelial cells. In both cell types, the viral protein activates NF-κB, a major regulator of inflammatory responses, and triggers the NLRP3 inflammasome signaling pathway, as a first line innate immunity sensor. All of this is paralleled by the synthesis and release of soluble pro-inflammatory cytokines and an imbalanced production of coagulation regulators.

Hyperinflammation is a key feature of severe COVID-19, where monocytes play a crucial role in the complications driven by the disease [37]. Here we identify S protein as a direct stimulator of monocyte activation by triggering the NF-κB pathway and releasing cytokines such as IL-6, which is elevated in the circulation of COVID-19 patients where it correlates with T cell depletion [37]. In human monocytes, S protein also stimulates the expression of NLRP3 inflammasome components driven by NF-κB [38] and causes the assembly of the active complex leading to the generation and release of active IL-1β. We observed that not only monocytes, but also human endothelial cells released NLRP3 inflammasome-derived IL-1β when challenged with the viral S protein. In this cell type the presence of basal levels GSDMD-NT, which were not further stimulated by S protein or IL-1β, suggest that cell membrane pores may be available for the release of IL-1β to the extracellular space. However, other mechanisms for exporting IL-1β cannot be discarded since inflamed endothelial cells release extracellular vesicles which contain cytokines and other pro-inflammatory mediators [39]. In monocytes and endothelial cells extracellular IL-1β activates itself the NLRP3 inflammasome [30]. Thus, by promoting the synthesis and release of IL-1β, the viral S protein initiates an auto-inflammatory loop that amplifies the local production of the cytokine by different cell types. In terms of pathophysiology, the over-activation of the NLRP3 inflammasome in vascular cells has been tightly associated with vascular diseases, such as atherosclerosis, stroke or hypertension, and, more recently, with COVID-19-associated vasculopathy and hyperinflammation [40, 41].

Overall, the SARS-CoV-2 S protein as an isolated element can be sensed by the cellular innate immune system to produce active IL-1β. Importantly, this pro-inflammatory cytokine has revealed itself as a pivotal player in human vascular disease and atherosclerosis. The CANTOS trial demonstrated that specifically targeting IL-1β with the monoclonal antibody canakinumab reduced chronic low-grade inflammation and the incidence of cardiovascular events, independently of other factors such as hyperlipidemia [42]. Thus, the local production and release of IL-1β from human vascular cells and monocytes stimulated by the S protein may contribute to vascular dysfunction in the context of COVID-19, favoring vascular inflammation and perhaps amplifying and aggravating pre-existing vascular lesions.

In a close relation with hyperinflammation, hypercoagulation and thrombotic events are acknowledged as major complications in COVID-19 and post-COVID-19 patients [43, 44], and they represent rare but challenging adverse effects of S protein mRNA-based vaccines [45]. Interestingly, post-vaccine complications have been recently related to elevated levels of circulating levels of S protein [46]. In this pathological context, vWF, a key coagulation factor formed within endothelial cells and megakaryocytes, has been repeatedly reported elevated in the circulation of COVID-19 patients [47, 48], where it acts as a marker of endotheliopathy and a predictor of poor outcome [49].

Once released, vWF can assemble into filamentous multimers with a high coagulant activity that promote platelet adhesion and aggregation [50]. To avoid excessive thrombogenic activity, the protease ADAMST-13, a physiological regulator of hemostasis, trimers the vWF multimers into smaller and less active molecules [50]. Here, we observed that while both S protein and IL-1β augmented the endothelial content of vWF and its release to the extracellular space, only the cytokine was capable of a parallel induction of ADAMTS-13 in order to counteract the pro-coagulant capacity of vWF. In other words, in human endothelial cells the viral S protein evokes a disbalance in the vWF:ADAMTS-13 ratio which may favor thrombi formation, similar to that observed in certain pro-coagulant and pro-thrombotic conditions such as thrombotic thrombocytopenic purpura or stroke [50, 51]. Indeed, lower ADAMS-13 levels have been described in COVID-19 patients where an elevated vWF:ADAMST-13 ratio strongly correlates with the severity of the disease and associates with endotheliopathy and immune dysfunction in long COVID syndrome [52–54].

In parallel, S protein increased the endothelial content of other factors involved in coagulant responses such as FVIII and TF, that are equally associated with hypercoagulability in COVID-19 patients [55–57]. The latter was also over-expressed in monocytes, where the S protein behaved similarly to a series of pro-inflammatory cytokines (IL-1β, IL-6, and TNF-α) massively released in COVID-19 that induce TF expression in leukocytes and non-immune cells, favoring a hypercoagulable state and thrombus formation [56].

**Fig. 6:**
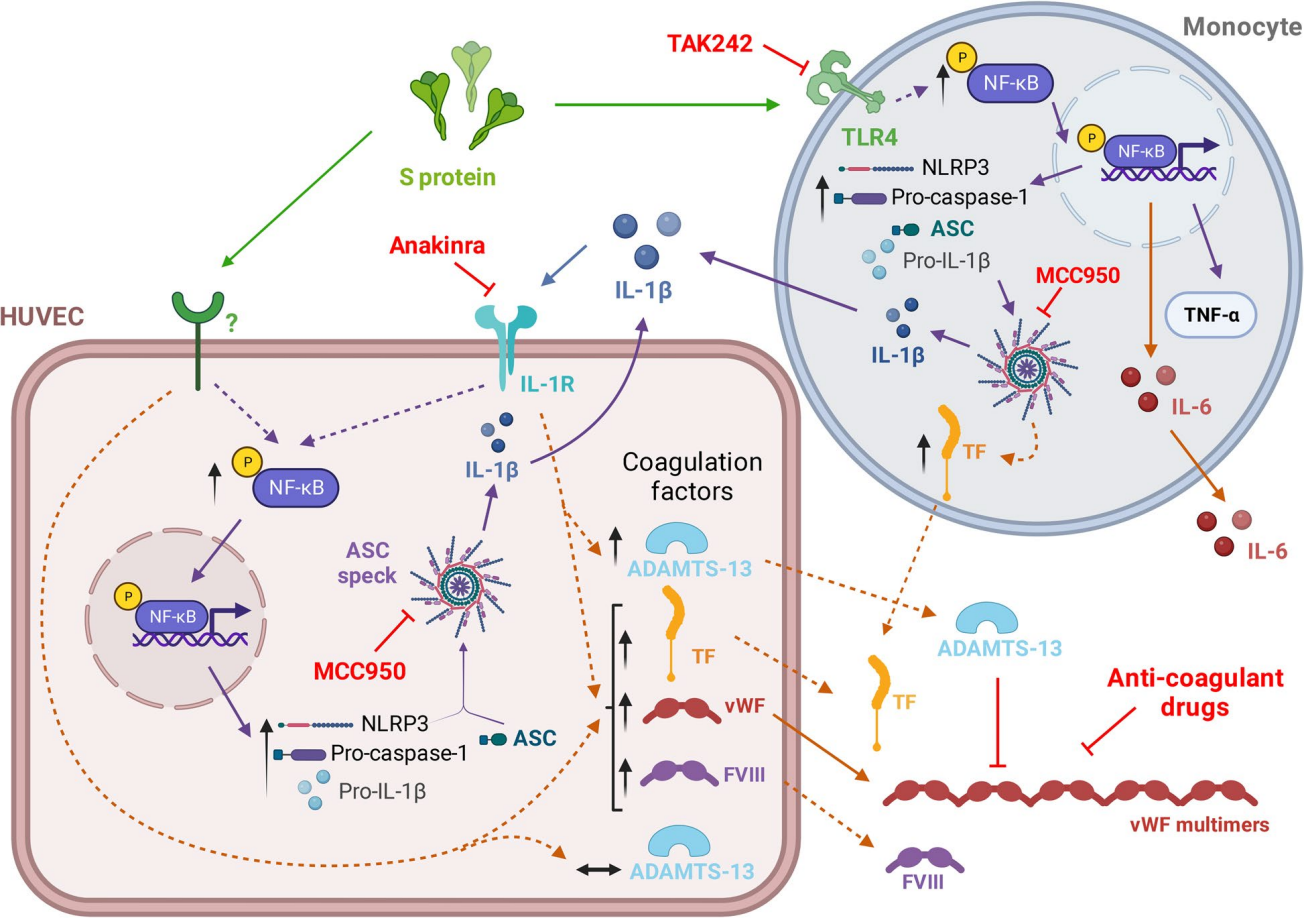
Diagram of cellular pathways activated by SARS-CoV-2 S protein and the targets for pharmacological interference. In human monocytes and HUVEC, S protein as an isolated element activates NF-κB, promotes the release of pro-inflammatory cytokines and triggers the priming and activation of the NLRP3 inflammasome system, leading to the formation and release of mature IL-1β. This cytokine can in turn act on IL-1R, thus fueling and amplifying an auto-inflammatory loop. Moreover, S protein enhances the cellular content of factors involved in coagulation processes, including vWF, FVIII and TF. In human endothelial cells, S protein fails to over-express the protease ADAMS-13 in order to counteract the hypercoagulation capacity of vWF multimers. While these effects of S protein are mediated by TLR4 in human monocytes, the receptors involved in HUVEC remain to be elucidated. Drugs such as anakinra, TAK242 (resotorvid), MCC950, or anti-coagulant drugs could interfere with the deleterious pro-inflammatory and pro-coagulant actions of S protein

The pharmacological inhibition of the NLRP3 inflammasome activation or the blockade of IL-1R tended to attenuate the endothelial over-expression of coagulation factors induced by the S protein. In monocytes, NLRP3 inflammasome is known to mediate TF release, which is a primary initiator of the coagulation cascade [58]. Thus, an intimate relationship seems to exist between the pro-inflammatory and pro-coagulant activities of the S protein of the SARS-CoV-2 crown, which opens the field for different pharmacological interventions to interfere with the deleterious activation exerted by such an isolated SARS-CoV-2 element on human vascular and immune cells (Fig. 6). In this line, a recent study unveiled a spontaneous NLRP3 inflammasome over-activation and IL-1β secretion in monocytes from severe COVID-19 patients that could be reverted by treating the patients with the IL-1 receptors blocker anakinra [59].

The activation of the NLRP3 inflammasome was achieved by the whole S protein but not by its isolated S1 fragment which contains the domain binding region (DBR) that interacts with host receptors. While this contradicts some pre-existing studies reporting a functional role for S1 [29, 60, 61], our observation suggests that the homotrimeric S1 structure or even the whole S protein is required to interact with cellular receptors triggering intracellular signaling that leads to the activation of human endothelial and immune cells. Indeed, the whole S protein is the main product of mRNA vaccines developed against COVID-19 [62, 63]. By directly activating immune and endothelial cells from a certain concentration threshold, the S protein could be at the basis of adverse effects related to COVID-19 vaccination, especially in subjects with reported or subclinical endothelial dysfunction or immune disbalance.

Although ACE2 was early identified as a main cell receptor interacting with the S protein of the SARS-CoV-2 corona, it was not detectable in the primary human monocyte cultures used in this study, in line with previous reports showing highly restricted expression of ACE2 primary human immune cells [64, 65]. A similar finding was made in primary endothelial cultures, a cell type for which controversial reports exists regarding the presence or not of ACE2 [66, 67].

Other receptors may favour the recognition and interaction of S-protein with host cells, including toll-like receptor 4 (TLR4), basigin (CD147), and dipeptydilpeptidase-4 (CD26) [7, 64, 68]. Monocytes present constitutive surface expression of TLR4, which is the canonical receptor implicated in the recognition of as bacterial lipopolysaccharides and has been implicated in various diseases [69–71]. Ligand binding to TLR4 leads to its oligomerization which in turn can activate myeloid differentiation factor 88 (MYD88) pathway, culminating in the activation of transcription factor NF-κB [72]. In such a context, blocking TLR4 signalling, which has been proposed as a possible therapeutic approach in COVID-19 patients [73], arises as a relevant option to attenuate the direct actions of the S protein as an isolated viral element. Moreover, the fact that TLR4 did not mediate the direct actions of the S protein in endothelial cells underpins the diversity and complexity of the SARS-CoV-2-host interactions and demands further research for better understanding the interactions of the viral protein with a key vascular component such is the endothelium.

Beyond acute COVID-19 episodes, S protein could play a role in the context of COVID sequalae that have been recently associated to persisting circulating levels of the protein [74]. Moreover, SARS-CoV-2 reservoirs have been detected in tissues of post-COVID-19 patients [75]. Years after SARS-CoV-2 viral infection, the S protein, with no other parts of the virus, has been found in organs like the brain [76] in association with persistent local inflammation and vascular damage. Although these studies did not recruit patients diagnosed with long COVID-19, we hypothesize that a non-resolved and sustained endothelial and immune inflammation together with hypercoagulation and thrombosis mediated by the S protein might be a contributor of long-term sequalae. Since blood vessels traverse every organ and immune cells are present in every tissue, both cell types can be key unifying players in a wide variety of prolonged symptoms of the disease.

Taken together, the findings of the present highlight the role of SARS-CoV-2 S protein as an ethiopatogenic agent in the clinical manifestations in acute or long COVID-19 and raises opportunities for novel pharmacological interventions based on S protein blockade, NLRP3 inhibition, monoclonal antibodies or fusion proteins against IL-1β or IL-1R and TLR4 antagonists, among other, together with anticoagulant therapies.

In conclusion, the S protein from SARS-CoV-2 acts as a unifying stimulus directly promoting pro-inflammatory and pro-coagulant activation of human immune and endothelial cells. Interfering with the S protein-host receptor binding or attenuating the deleterious signaling triggered by this isolated viral element might provide therapeutical approaches to confront COVID-19 vaccine-derived complications or acute and long-term complications of the disease.

### Supplementary Information

**Additional file 1.**

**Additional file 2.**

## Data Availability

All data generated and analyzed during the current study are available from corresponding author on reasonable request.

## Abbreviations

COVID-19: coronavirus disease 2019
SARS-CoV-2: severe acute respiratory syndrome corona virus 2
S protein: spike protein
RBD: receptor binding domain
ACE2: angiotensin converting enzyme 2
TLR4: toll-like receptor 4
CD: cluster differentiation
vWF: von Willebrand factor
FVIII: factor VIII
TF: tissue factor
NLRP3: NLR family pyrin domain-containing 3
IL: interleukin
ASC: apoptosis-associated speck-like protein
HUVEC: human umbilical vein endothelial cells
PBMC: peripheral blood mononuclear cell
pp65: phosphorylated-p65
NF-κB: nuclear factor-kappa B
ADAMST-13: A disintegrin and metalloprotease with a thrombospondin type-1 motif, member 13
TNF-α: tissue necrosis factor alpha
RT-qPCR: real time quantitative polymerase chain reaction
siTLR4: specific TLR4 short interfering RNA
