# Correction: SARS-CoV-2 S protein activates NLRP3 inflammasome and deregulates coagulation factors in endothelial and immune cells

**DOI:** 10.1186/s12964-024-01491-3

**Published:** 2024-01-23

**Authors:** Alicia Villacampa, Enrique Alfaro, Cristina Morales, Elena Díaz-García, Cristina López-Fernández, José Luis Bartha, Francisco López-Sánchez, Óscar Lorenzo, Salvador Moncada, Carlos F. Sánchez-Ferrer, Francisco García-Río, Carolina Cubillos-Zapata, Concepción Peiró

**Affiliations:** 1 Department of Pharmacology, School of Medicine, Universidad Autónoma de Madrid, Madrid, Spain; 2 Respiratory Diseases Group, Respiratory Service, La Paz University Hospital, IdiPAZ, Madrid, Spain; 3 Biomedical Research Network- Ing Center On Respiratory Diseases (CIBERES), Madrid, Spain; 4 Department of Obstetrics and Gynecology, School of Medicine, Universidad Autónoma de Madrid, Madrid, Spain; 5 Gynecology and Obstetrics Service, La Paz University Hospital, Madrid, Spain; 6 Laboratory of Diabetes and Vascular Pathology, IIS-Fundación Jiménez Díaz, Madrid, Spain; 7 Biomedical Research Networking Centre On Diabetes and Associated Metabolic Disorders (CIBERDEM), Madrid, Spain; 8 Department of Medicine, School of Medicine, Universidad Autónoma de Madrid, Madrid, Spain; 9 Vascular Pharmacology and Metabolism (FARMA- VASM) Group, IdiPAZ, Madrid, Spain

**Correction: Cell Commun Signal 22, 38 (2024)**

**https://doi.org/10.1186/s12964-023-01397-6**

Following publication of the original article [1], it was identified that Carolina Cubillos-Zapata was not marked as a co-corresponding author. The publishers apologize for the error.

The author group has been updated and the original article [1] has been corrected.
