# Supplementary material for: SARS-CoV-2 S protein activates NLRP3 inflammasome and deregulates coagulation factors in endothelial and immune cells
Source: Cell Commun Signal. 2024 Jan 15;22:38. doi: 10.1186/s12964-023-01397-6 (PMC10788971; doi:10.1186/s12964-023-01397-6)
Supplement: Supplementary file 2 — Additional file 1. [file 12964_2023_1397_MOESM1_ESM.docx]

**Supplemental Files**

**SARS-CoV2 S protein activates NLRP3 inflammasome and deregulates coagulation factors in endothelial and immune cells**

by

Alicia Villacampa^1,*^, Enrique Alfaro^2,3,*,^ Cristina Morales^1^, Elena Díaz-García^2,3^, Cristina López-Fernández^2^, José Luis Bartha^4,5^, Francisco López-Sánchez^5^, Óscar Lorenzo^6,7, 8^, Salvador Moncada^1^, Carlos F. Sánchez-Ferrer^1,9^, Francisco García-Río^2,3,8^, Carolina Cubillos-Zapata^2,3^ and Concepción Peiró^1,9^

^1^Department of Pharmacology, School of Medicine, Universidad Autónoma de Madrid, Spain; ^2^Respiratory Diseases Group, Respiratory Service, La Paz University Hospital, IdiPAZ, Madrid, Spain; ^3^Biomedical Research Networking Center on Respiratory Diseases (CIBERES), Madrid, Spain; ^4^Department of Obstetrics and Gynecology, School of Medicine, Universidad Autónoma de Madrid, Madrid, Spain; ^5^Gynecology and Obstetrics Service, La Paz University Hospital, Madrid, Spain; ^6^Laboratory of Diabetes and Vascular pathology, IIS-Fundación Jiménez Díaz, Madrid, Spain; ^7^Spanish Biomedical Research Centre on Diabetes and Associated Metabolic Disorders (CIBERDEM) Network, Madrid, Spain; ^8^Department of Medicine, School of Medicine, Universidad Autónoma de Madrid, Madrid, Spain; ^9^Vascular Pharmacology and Metabolism (FARMAVASM) group, IdiPAZ, Madrid, Spain

*A.V. and E.A. contributed equally to this study.

‡Authors for correspondence: Concepción Peiró, Department of Pharmacology, Faculty of Medicine, Universidad Autónoma de Madrid 28029 Madrid, Spain; email: concha.peiro@uam.es; Carolina Cubillos-Zapata, Respiratory Diseases Group, Respiratory Service, La Paz University Hospital, IdiPAZ, Madrid, Spain; email: cubilloszapata@gmail.com

**Supplementary Methods**

**Materials**

M199 culture medium, fetal calf serum (FCS) were purchased from Biological Industries (Beit-Hamek, Israel). RPMI1640 culture medium was purchased from ThermoFisher Scientific (Waltham, MA, USA). Heparin, endothelial cell growth supplement (ECGS), amphotericin, type II collagenase, type I collagen, EDTA, sodium orthovanadate, phenyl-methylsulfonyl fluoride (PMSF) and the sodium salt CP-456773 (also known as MCC 950) were purchased from Sigma (St. Louis, MO, USA). SARS-CoV2 spike (S) recombinant protein was purchased from BioTechne (Minneapolis, MN, USA), while human recombinant IL-1β was from from Preprotech (London, UK). Anakinra and TAK242 and MCC950 were obtained from Biovitrum (SOBI, Stockholm, Sweden) and Sigma, respectively.

**Human umbilical vein endothelial cells culture**

HUVEC were isolated by chemical digestion with type II collagenase (2 mg/mL), and cultured in M199 medium with 20 % FCS, 25 μg/mL ECGS, 100 μg/mL heparin and antibiotics (100 U/mL penicillin, 100 µg/mL streptomycin and 2.5 µg/mL amphotericin B) at 37°C in a humidified atmosphere with 5 % CO_2_. For all experiments, cells at passages 1–5 were treated for 18-24 h.

**PBMCs, monocytes isolation, and cell cultures**

Twenty milliliters of blood were taken from peripheral vein from healthy subjects (aged 18-65) by venipuncture into EDTA tubes. Blood was layered on top of 10 mL Ficoll-Paque Plus (Amersham Biosciences) and centrifuged at 1500 rpm for 20 minutes at 24°C. Plasma was removed from the upper layer. Then, the peripheral blood mononuclear cells (PBMCs) were removed from the interphase and washed in PBS.

The enriched monocytes cultures were obtained by adherence. Briefly, PBMCs were placed on m6 plates (5x10^5^ monocytes/well) in 1 mL RPMI 1640 medium supplemented with 1% penicillin/streptomycin. After 45 minutes medium including non-adherent cells is withdrawn leaving enriched monocytes culture in the plate. 0.5 mL of medium supplemented with 10% owns donor plasma is added to the cells and cultured for 3 or 16h. After culture, enriched monocytes’ supernatant was removed, centrifuged to eliminate any cell in suspension. Adherent cells were rinsed with PBS twice, then carefully harvested using cellular scratcher and lastly prepared for flow cytometry or RNA extraction. Enriched monocytes that were used for protein extraction were not scratched, instead protein extraction with RIPA buffer was performed directly on the plate at 4ºC for 45 minutes.

**SARS-Cov2 S protein stimulation of HUVEC and monocytes**

HUVEC were stimulated with a concentration range of 7-70 nM recombinant SARS-CoV-2 Spike protein. For selected experiments a single submaximal concentration of 35 nM and 15 nM was used for HUVEC and monocytes, respectively. The treatment times are indicated in the corresponding figure legends.

**Western Blot**

HUVEC or enriched monocytes were lysed and the protein content in cell lysates was quantified by the bicinchoninic acid (BCA) method (Thermo Fisher Scientific, Illinois, USA). Thereafter, 20 μg of protein lysates were separated by SDS-PAGE electrophoresis and transferred to polyvinyl membranes (Merck, Darmstadt, Germany). Proteins were detected as previously described [1]. Primary antibodies against NLRP3 (20B-0012; Adipogene, Switzerland; 1:1,000), IL-1β/IL-1F2 (AF-401-NA; R&D System, USA; 1:1,000), phospho-p65 (p-p65; (S536); Cell Signaling, USA; 1:1,000), p65 (B14E12; Cell Signaling, USA; 1:1,000), TF (sc-374441, Santa Cruz; 1:1,500), Factor VIII (ab275373, Abcam; 1:1,000), vWF (A0082, Dako; 1:1,000) and GSDMD (HPA044487, Sigma-Aldrich; 1:500) were used, followed by incubation with corresponding horseradish peroxidase-conjugated secondary antibodies (Bio-Rad; 1:10,000). Protein levels were normalized to β-actin signal (Sigma-Aldrich; 1:10,000). Immunoreactive bands were detected using an enhanced chemiluminescence ECL detection kit (Bio-Rad, California, USA) and quantified by densitometry using ImageJ 1.51w free software.

**Visualization of NLRP3 activation by indirect immunofluorescence**

After incubation with a primary anti-ASC antibody (ADI-905-173; Enzo Life Science, Switzerland; 1/250), followed by an Alexa Fluor 647-conjugated goat anti-rabbit IgG secondary antibody (Jackson Immuno Research, Cambridge, UK). Nuclei were counterstained with 1 μmol/L 4’-6’-diamidino-2-phenylindole (DAPI) (Molecular Probes-Invitrogen Corporation; USA). The number of ASC specks per field was quantified by manual blind scoring of 27 radial distributed fields per sample under an inverted microscope Eclipse TE300 (Nikon). Representative images were acquired with a Leica TCS SPE confocal microscope using 63 X oil immersion objective (Leica, Wetzlar, Germany).

**Quantification of vWF and IL-1β secretion by ELISA in HUVEC**

The supernatants of HUVEC challenged with selected stimuli and or inhibitors we collected, centrifuged to remove cell debris and stored at -80ºC until determination of human vWF and IL-1β by using commercial kits from Sigma-Aldrich and Invitrogen (IL-1β Human ELISA Kit, High Sensitivity, BMS224HS), respectively, according to the manufacturer instructions.

**mRNA isolation and quantification**

Total RNA was extracted from endothelial cells, monocytes, and renal HK2 by using TRIzol reagent (ref: 15596026, Invitrogen, MA, USA), following the manufacturer's instructions. The concentration and integrity of the extracted RNA were assessed using the Nanophotometer® N60 (IMPLEN, München, Germany). Subsequently, cDNA synthesis was retro-transcripted by using 1.5 µg of RNA and the High-Capacity cDNA Reverse Transcription Kit (ref: 4368813, Applied Biosystems, MA, USA) in the Veriti Thermal Cycler (ThermoFisher Scientific). Then, gene expression was evaluated by quantitative PCR (qPCR) by using TaqMan® or SYBR-green® technologies (Applied Biosystems). For the former, cDNA (30-100 ng) from cells was mixed with the universal qPCR master mix (Taq-Man™ Universal PCR Master Mix, ref:4318157, Applied biosystems) and with the expression assay for *ACE2* (Hs01085333_m1, FAM-fluorophore). Then, the qPCR was performed in the StepOnePlus™ Real-Time PCR System (ThermoFisher Scientific). Each sample was run in triplicate and internal variations higher than 0.3 cycles were not considered. The relative gene expression was revealed by the comparative ΔΔCт method (StepOne™ Plus v.2.3 software). As a housekeeping gene, we used the eukaryotic ribosomal 18s labeled with VIC-fluorophore (Hs99999901_s1; ThermoFisher Scientific). For the later, cDNA (50 ng) was mixed with NZY Supreme qPCR Green MasterMix (Nzytech, Lisboa, Portugal) and specific primers for targeted genes synthesized by Eurofins Genomics Srl (Vimidrone, Italy) (Supplementary Table 1). The mRNA expression was also normalized to the ribosomal 18S expression.

In addition, the amount of potential *ACE2*-mRNA messengers was confirmed by digital PCR (dPCR). The dPCR reaction mixture was prepared following the standard QuantStudio Absolute Q dPCR protocol, with 10 µl of the Absolute Q DNA Digital PCR Master Mix (ref: A52490, Applied Biosystems, MA, USA), the Taqman® gene expression assay (*ACE2*: Hs01085333_m1, FAM-fluorophore), and the input cDNA (30-100 ng). Each 9 µl reaction mixture was loaded into a 24k micro chamber QuantStudio MAP16 Plate (ref: A52865, Applied Biosystems, MA, USA), followed by the addition of 15 µl of isolation buffer (ref: A52730, Applied Biosystems, MA, USA). All samples were loaded in duplicate in the QuantStudio™ Absolute Q™ Digital PCR System (Thermo Fisher, MA, USA). The dPCR run consisted of a 96°C preheat reaction of 10 min, followed by 40 cycles of denaturation at 95°C for 5 sec, and annealing and extension at 60°C for 15 sec. The Absolute Q Digital PCR Software (Applied Biosystems v6.2.1) and a 95% confidence interval were used to assess the transcript copies/µl of *ACE2*. The ROX dye was employed as an internal control for fluorescence normalization in the detection channels.

**Flow cytometry and inflammatory cytokines analysis in monocytes**

Cells were fixed following a standard protocol using the Transcription Factor Buffer Set (Becton-Dickinson Biosciences). Cells were labeled (30 minutes, 4ºC) with specific antibodies against CD14 (14A-100T, Inmunostep), NLRP3 (130-111-209, MACS Miltenyi Biotec), ASC (653904, Biolegend). Active caspase-1 was determined by flow cytometry using FAM-FLICA Caspase-1 assay (Immunochemistry Technologies, California) following manufacturer’s instructions. Cells were acquired by BD FACS-Calibur flow cytometer (Becton-Dickinson Biosciences, Belgium), and data were analyzed using FlowJo vX.0.7 software (FlowJo, USA).

The inflammatory cytokines, IL-1β and IL-6, concentration was measured using BD Human Inflammatory Cytokine Citometric Bead Array kit (551811, Becton-Dickinson Biosciences), acquired by BD FACS-Calibur flow cytometer (Becton-Dickinson Biosciences) and analyzed by FCAP Array software (Becton-Dickinson Biosciences).

**TLR4 and NLRP3 Inhibition Assays**

To inhibit TLR4, TAK242 (resatorvid; 5µM, Sigma-Aldrich) was added to the culture and cells were maintained for 3 or 16-18 hours, following a similar approach to previously published [2]. For NLRP3 inflammasome inhibition, MCC950 (1-5 µM; Sigma-Aldrich) was added to cell cultures for 16-18 hours, following the directions of previous publications [3, 4]. Moreover, the enriched monocytes were transfected with small interfering RNA (siRNA) specifically designed against TLR4 (Ambion; cat. #4390824, siRNA ID #s14194) using the Human Monocyte Nucleofector kit (Lonza, cat. #VPA-1007). In short, 10^6^ cells were centrifuged and resuspended in 100uL transfection buffer including or not 100 nM of TLR4 siRNA. Cell suspension was rapidly introduced in electroporation cuvette and transfected following manufacturer’s directions. Then, cells were transferred to 900uL pre-warmed RPMI 1640 medium supplemented with 10% plasma from the cell’s donor. Cells were cultured for 3 hours at 37°C with 5% CO_2_ and afterwards were stimulated with S protein and cultured for 16 hours more.

**Supplementary Tables**

**Supplementary Table 1.** qPCR Primer sequences used in the study.

| NF-κB | Forward primer | GGTGCGGCTCATGTTTACAG |
| --- | --- | --- |
|  | Reverse primer | GATGGCGTCTGATACCACGG |
| TNF-α | Forward primer | GGCGTGGAGCTGAGAGATAAC |
|  | Reverse primer | GGTGTGGGTGAGGAGCACAT |
| IL-6 | Forward primer | GGTACATCCTCGACGGCATCT |
|  | Reverse primer | GTGCCTCTTTGCTGCTTTCAC |
| NLRP3 | Forward primer | TGCCCGTCTGGGTGAGA |
|  | Reverse primer | CCGGTGCTCCTTGATGAGA |
| ASC | Forward primer | AACCCAAGCAAGATGCGGAAG |
|  | Reverse primer | AACCCAAGCAAGATGCGGAAG |
| Casp-1 | Forward primer | GGAAACAAAAGTCGGCAGAG |
|  | Reverse primer | ACGCTGTACCCCAGATTTTG |
| TF | Forward primer | CCCAAACCCGTCAATCAAGTC |
|  | Reverse primer | CCAAGTACGTCTGCTTCACAT |
| 18S | Forward primer | CGGCGACGACCCATTCGAAC |
|  | Reverse primer | GAATCGAACCCTGATTCCCCGTC |

**Ssupplementary Figures**

**
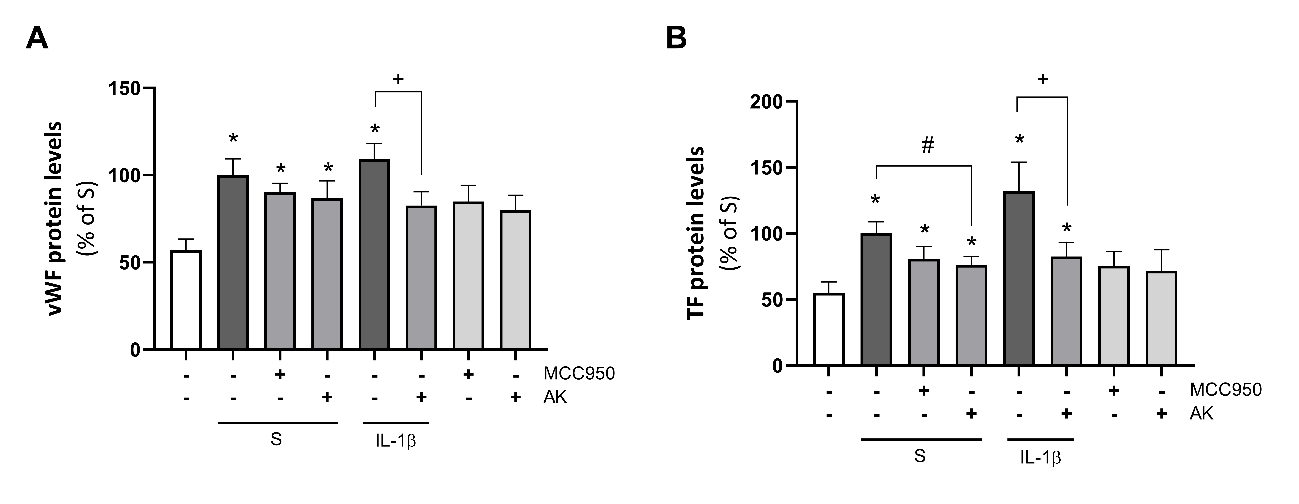
**

**Supplementary Figure 1. The IL-1R blocker anakinra prevents the induction of TF by the viral S protein in HUVEC.** Human umbilical vein endothelial cells (HUVEC) were treated with viral S protein (35 nM) alone or in the presence of the NLRP3 inflammasome inhibitor MCC950 (1 µM) or the IL-1R anakinra (AK; 1 µg/mL). Some cultures were also treated with IL-1β (2.5 ng/mL) with or without anakinra. In total cell lysates, the protein levels of (A) VWF (n=5-7) and (B) TF (n=8) were determined by Western blot with β-actin used as a loading control. Bar graphs represent mean ± SEM. Statistical differences were analyzed by t-test. *p < 0.05 versus control with no S protein; +p < 0.05 versus IL-1β; # p < 0.05 versus S.

**Supplementary Figure 2. Time-dependent expression of NF-κB in monocytes.** Enriched monocytes were culture under control condition or stimulated with the viral S protein (S15) for 1, 3 or 16 hours. (A) mRNA expression of NF-κB analyzed by RT-qPCR (n=9). (B) Representative image of phosphorylated p65 NF-κB particle Western blot band at a respective weight value of 65kDa from enriched monocytes lysate. (C) mRNA expression of TNF-α analyzed by RT-qPCR (n=9). (D) mRNA expression of IL-6 analyzed by RT-qPCR IL-6 (n=9). Differences were analyzed by two-way ANOVA and Sidak’s multiple comparisons test. Error bars: mean ± SEM. Statistically significant differences of S15 against control are stated: *p < 0.05.

**Supplementary Figure 3. Gating strategy for flow cytometry.** (A) Representative blots showing gating strategy for monocytes and comparative histogram for NLRP3 fluorescence intensity in control and S15 treatment. (B) Representative blots showing gating strategy for monocytes and comparative histogram for ASC fluorescence intensity in control and S15 treatment. (C) Representative blots showing FCS/SSC and gating strategy for monocyte’s positive for active Caspase-1 including comparison between control and S15 treatment.

**Supplementary Figure 4. Protein and mRNA expression of NLRP3 components in monocytes.** (A) Representative image of NLRP3 Western blot band at a respective weight value of 115kDa from enriched monocytes lysate. (B) mRNA expression in enriched monocytes cultured for 16h under control and S protein (S15) conditions of NLRP3 (n=14), ASC (n=11), casp-1 (n=16) and TF (n=16). Differences were analyzed by Wilcoxon’s paired test. (C) Representative image of TF Western blot band at a respective weight value of 45kDa from enriched monocytes lysate. (D) Enriched monocytes were stimulated or not with S15, treated or not with Ac-YVAD-cmk (YVAD) and cultured for 16h. Left: normalized amount of casp-1^+^ CD14^+^ cells analyzed by flow cytometry (n=6); right: supernatant IL-1β relative concentration measured by CBA (n=5). Differences were analyzed by repeated measures ANOVA and Tukey’s multiple comparisons test. All data are represented as mean ± Standard Error of the Mean (SEM). Only statistically significant differences are stated: *p < 0.05 and **p < 0.01.

**
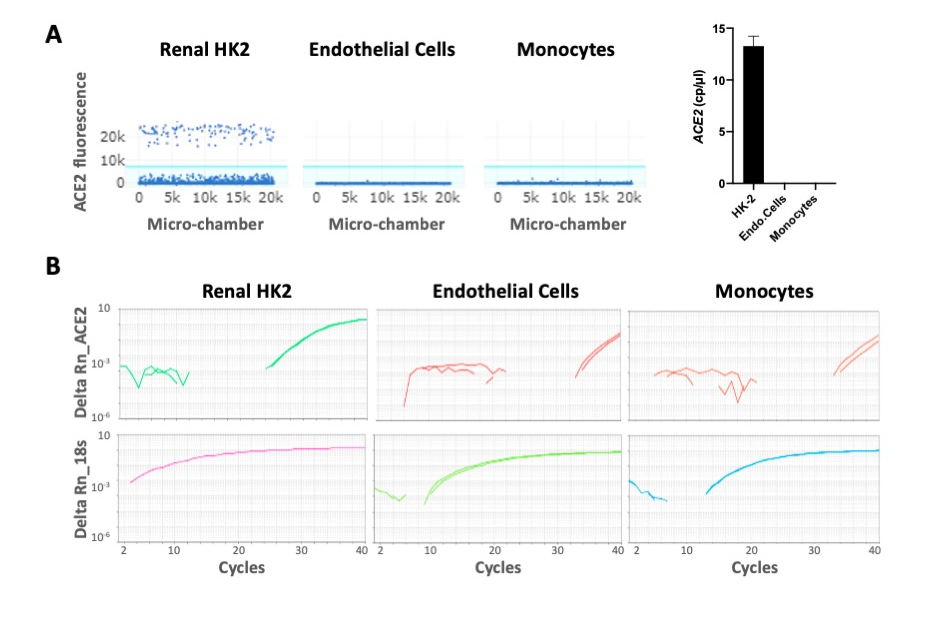
Supplementary Figure 5. Quantitative expression of ACE2 in vascular and renal cells.** (A) By dPCR, ACE2 transcripts were not detected in cultured endothelial cells (HUVEC) and monocytes, but it was in renal cells (HK2). (B) By qPCR, the ACE2 expression was not either detected (top panel) in cultured endothelial cells and monocytes though it was in HK2. The 18S ribosomal RNA was used as housekeeping gene.

**Supplementary Figure 6. NLRP3 components mRNA expression in monocytes abrogated by TAK242 or siTLR4.** (A) Enriched monocytes cultured under control, S15, TAK and S15-TAK conditions mRNA expression of NLRP3 (n=8), ASC (n=6), casp-1 (n=9) and TF (n=9). (B) mRNA expression of NLRP3 (n=4), ASC (n=3), casp-1 (n=4) and TF (n=4) from enriched monocytes stimulated with S15 or not and transfected in absence (control) or presence of TLR4 siRNA (siTLR4). Differences were analyzed by Friedman’s test and Dunn’s test for multiple comparisons. Error bars: mean ± SEM. Only statistically significant differences are stated: *p < 0.05.

**
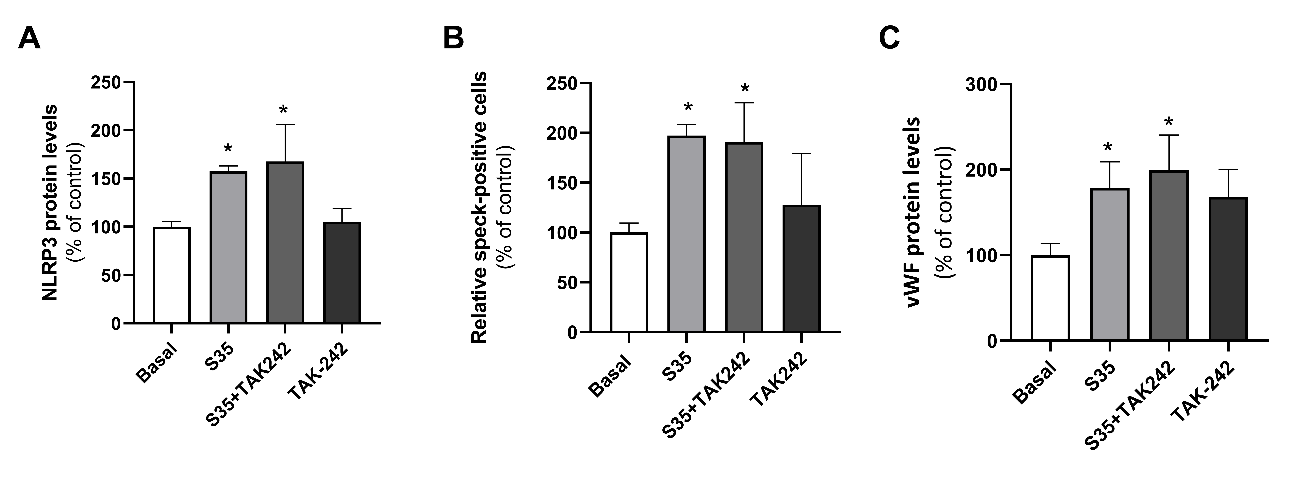
Figure S7. TLR4 receptor inhibition does not prevent NLRP3 inflammasome activation or VWF levels induction by S protein in HUVEC.** Cells were exposed to the S protein (35 nM) with or without the TLR4 inhibitor TAK242 (1 µM) for 18 h after which the following determinations were performed: (A) NLRP3 protein levels by Western blot in total cell lysates (n=4), (B) NLRP3 inflammasome activation as the number of ASC speck-positive cells determined by manual blind scoring of 27 radial distributed fields per sample (n=4), and (C) VWF protein levels by Western blot in total cell lysates (n=6-7). . For Western blots β-actin was used as a loading control. Bar graphs represent mean ± SEM. Statistical differences were analyzed by t-test. *p < 0.05 compared to basal.

**Supplementary References**

1. Romero A, San Hipolito-Luengo A, Villalobos LA, Vallejo S, Valencia I, Michalska P, Pajuelo-Lozano N, Sanchez-Perez I, Leon R, Bartha JL, et al: **The angiotensin-(1-7)/Mas receptor axis protects from endothelial cell senescence via klotho and Nrf2 activation.** *Aging Cell* 2019; **18:**e12913.

2. Xie H, Zhou H, Wang H, Chen D, Xia L, Wang T, Yan J: **Anti-beta(2)GPI/beta(2)GPI induced TF and TNF-alpha expression in monocytes involving both TLR4/MyD88 and TLR4/TRIF signaling pathways.** *Mol Immunol* 2013; **53:**246-254.

3. Diaz-Garcia E, Garcia-Tovar S, Alfaro E, Jaureguizar A, Casitas R, Sanchez-Sanchez B, Zamarron E, Fernandez-Lahera J, Lopez-Collazo E, Cubillos-Zapata C, Garcia-Rio F: **Inflammasome Activation: A Keystone of Proinflammatory Response in Obstructive Sleep Apnea.** *Am J Respir Crit Care Med* 2022; **205:**1337-1348.

4. Romero A, Dongil P, Valencia I, Vallejo S, Hipolito-Luengo AS, Diaz-Araya G, Bartha JL, Gonzalez-Arlanzon MM, Rivilla F, de la Cuesta F, et al: **Pharmacological Blockade of NLRP3 Inflammasome/IL-1beta-Positive Loop Mitigates Endothelial Cell Senescence and Dysfunction.** *Aging Dis* 2022; **13:**284-297.
