# Supplementary material for: SARS-CoV-2 S protein activates NLRP3 inflammasome and deregulates coagulation factors in endothelial and immune cells
Source: Cell Commun Signal. 2024 Jan 15;22:38. doi: 10.1186/s12964-023-01397-6 (PMC10788971; doi:10.1186/s12964-023-01397-6)

# Figure 1

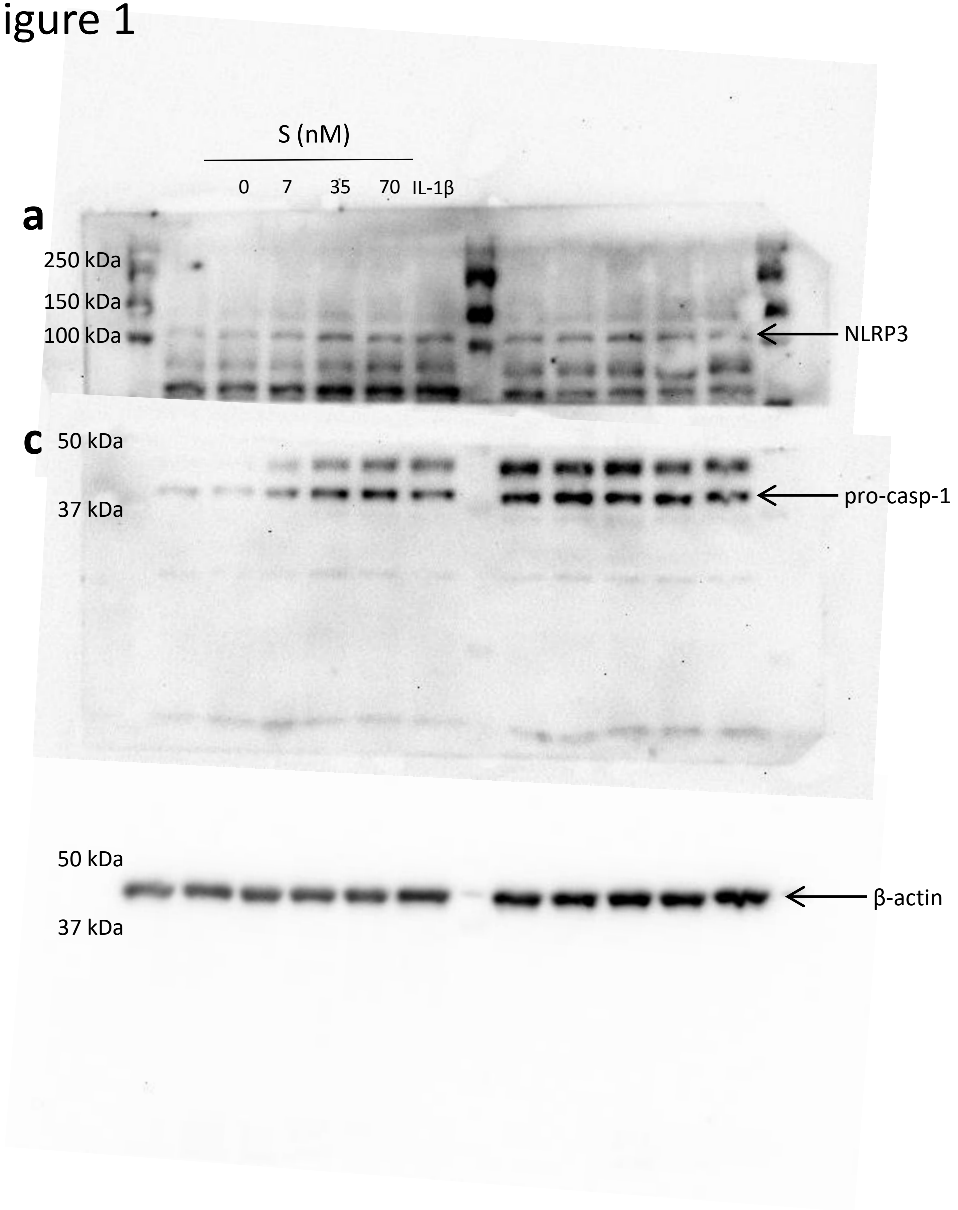

a
S (nM)
0
7
35
70
IL-1β
250 kDa
150 kDa
100 kDa
NLRP3
c
50 kDa
37 kDa
pro-casp-1
50 kDa
37 kDa
β-actin


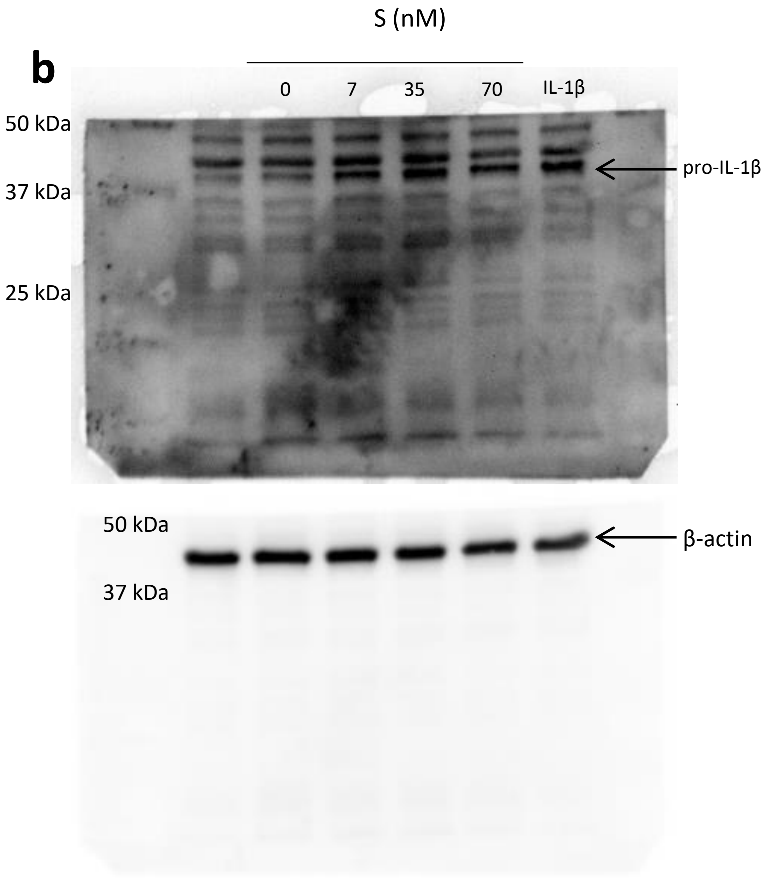

b
S (nM)
0
7
35
70
IL-1β
50 kDa
37 kDa
25 kDa
pro-IL-1β
50 kDa
37 kDa
β-actin

Figure 1

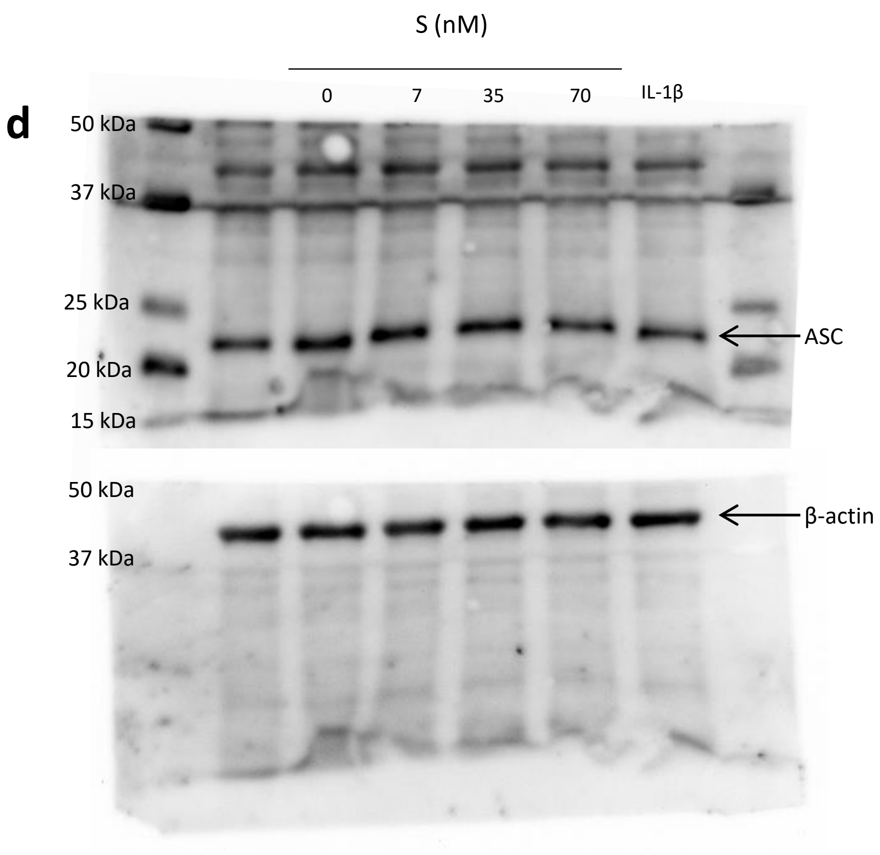
S (nM)
0
7
35
70
IL-1β
d
50 kDa
37 kDa
25 kDa
20 kDa
15 kDa
ASC
50 kDa
37 kDa
β-actin

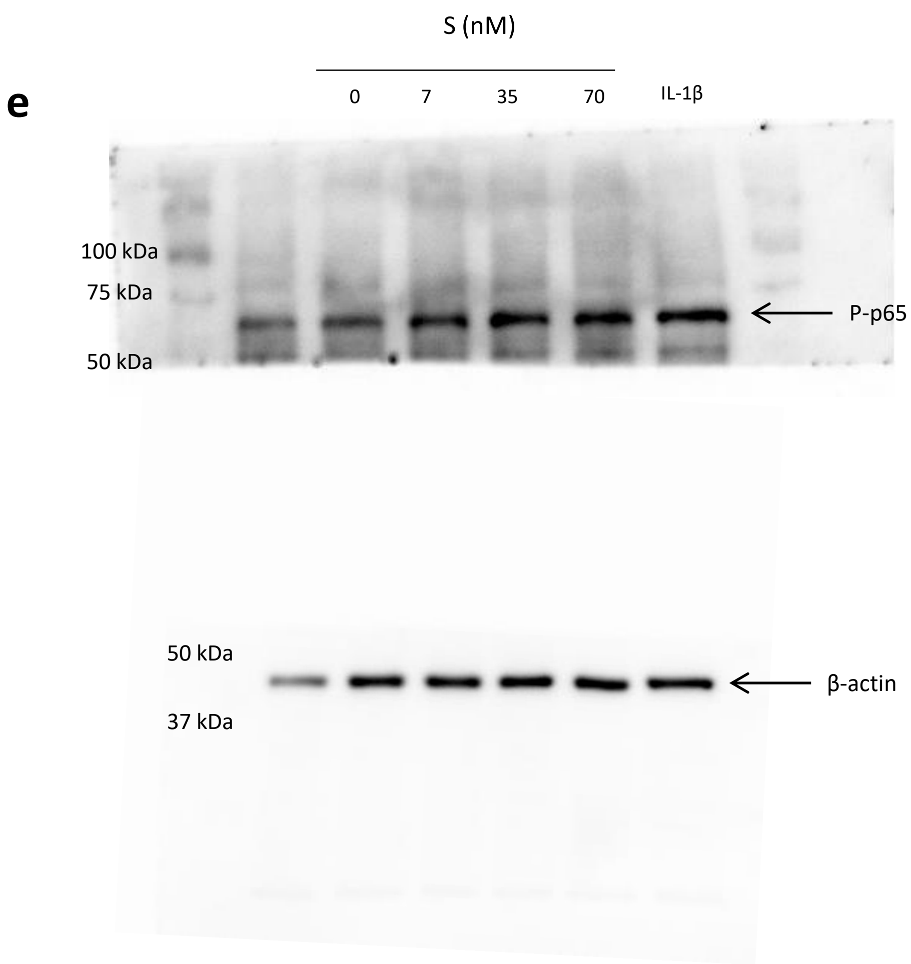
S (nM)
0
7
35
70
IL-1β
e
100 kDa
75 kDa
50 kDa
P-p65
50 kDa
37 kDa
β-actin

Figure 2

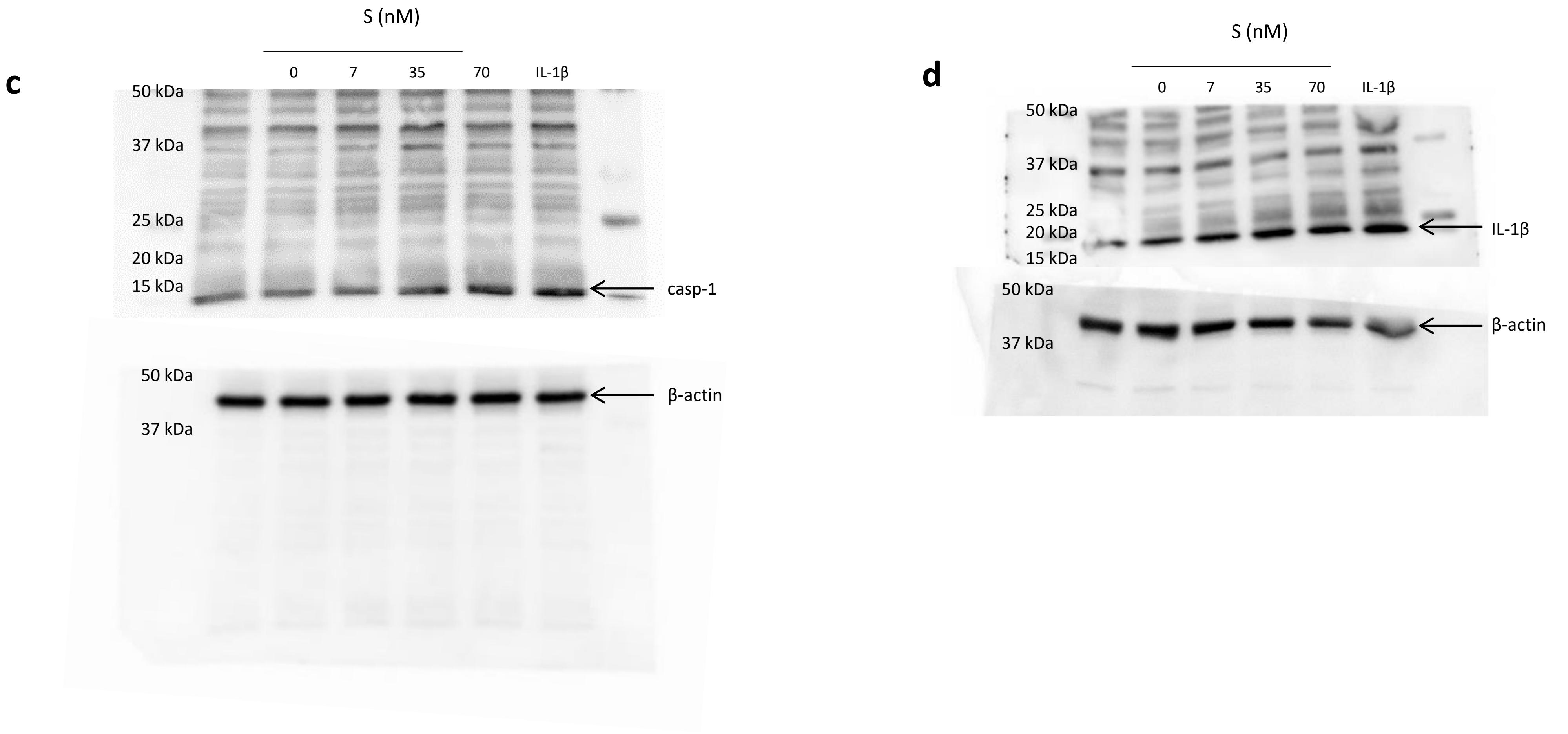

c
S (nM)
0
7
35
70
IL-1β
50 kDa
37 kDa
25 kDa
20 kDa
15 kDa
casp-1
50 kDa
37 kDa
β-actin
d
S (nM)
0
7
35
70
IL-1β
50 kDa
37 kDa
25 kDa
20 kDa
15 kDa
IL-1β
50 kDa
37 kDa
β-actin

Figure 2

**f**

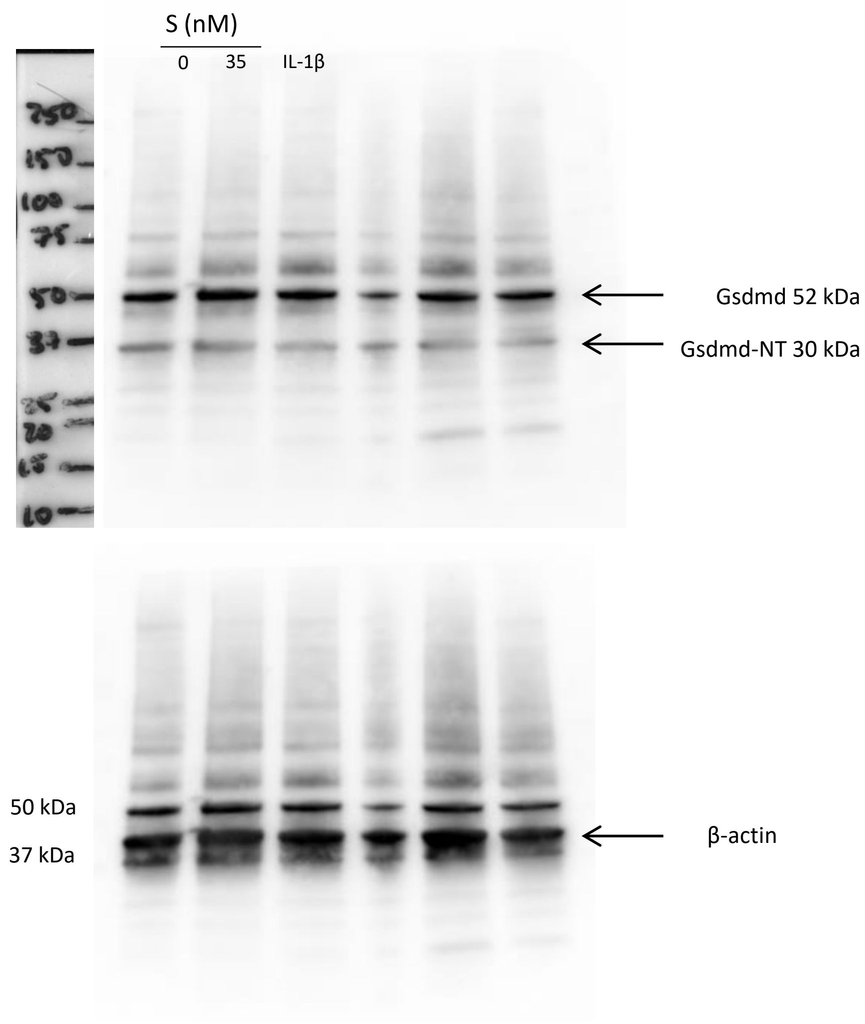
S (nM)
0
35
IL-1β
250
150
100
75
50
37
25
20
15
10
Gsdmd 52 kDa
Gsdmd-NT 30 kDa
50 kDa
37 kDa
β-actin

# Figure 3

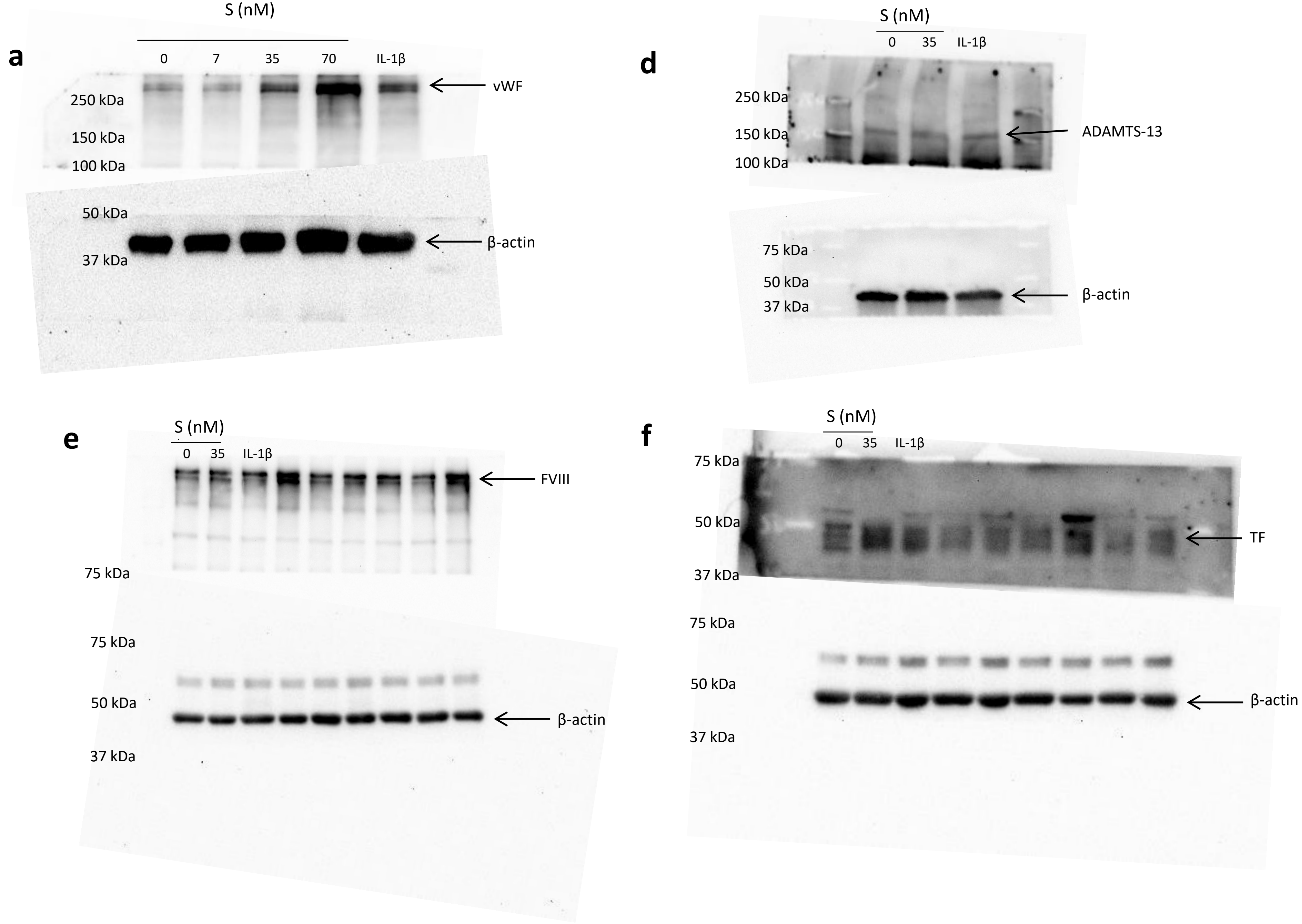

S (nM)
a
0
7
35
70
IL-1β
vWF
250 kDa
150 kDa
100 kDa
50 kDa
β-actin
37 kDa
S (nM)
d
0
35
IL-1β
250 kDa
150 kDa
100 kDa
ADAMTS-13
75 kDa
50 kDa
37 kDa
β-actin
e
S (nM)
0
35
IL-1β
FVIII
75 kDa
75 kDa
50 kDa
β-actin
37 kDa
f
S (nM)
0
35
IL-1β
75 kDa
50 kDa
TF
37 kDa
75 kDa
50 kDa
β-actin
37 kDa

Suplemmentary Figure 2B

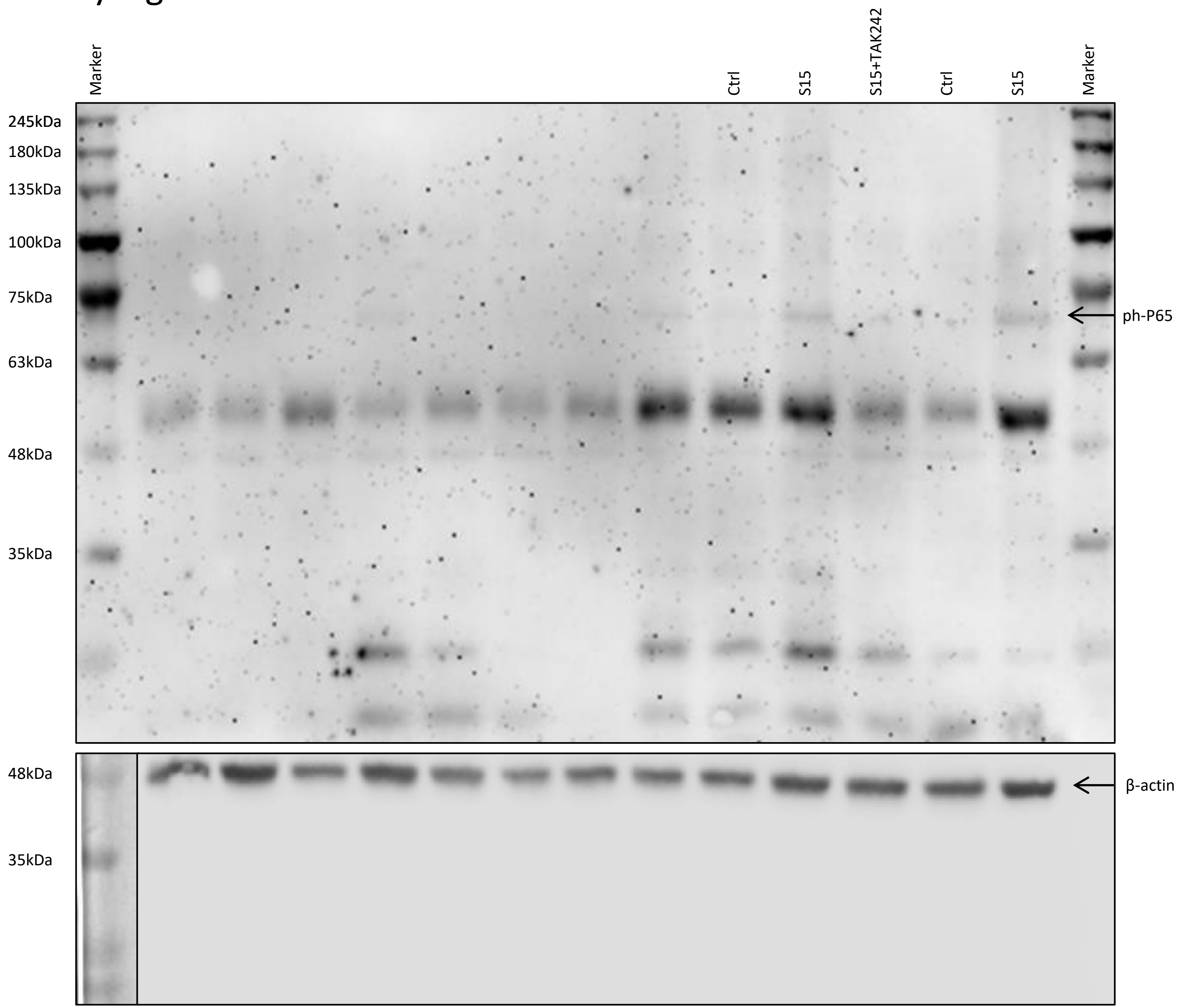

# Suplemmentary Figure 4A

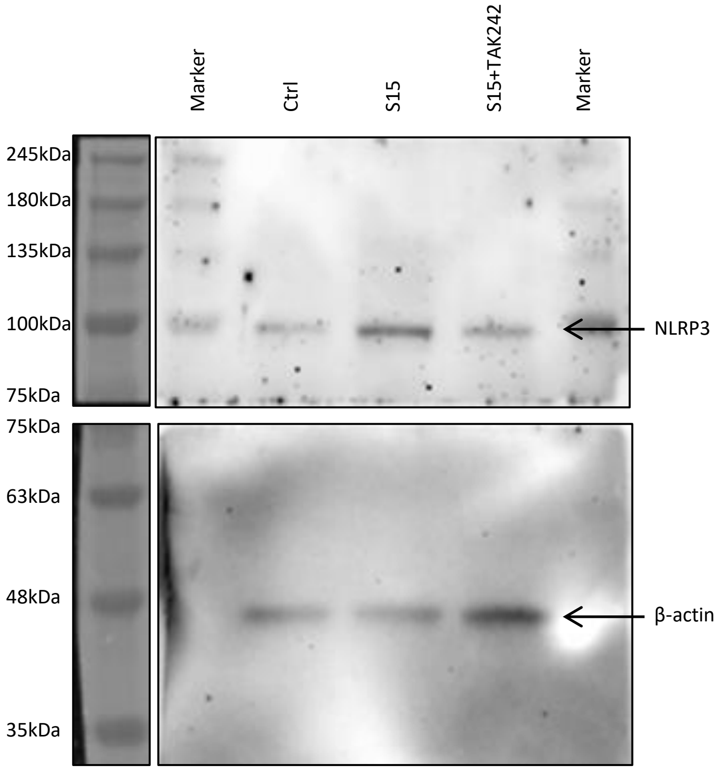


# Suplemmentary Figure 4C

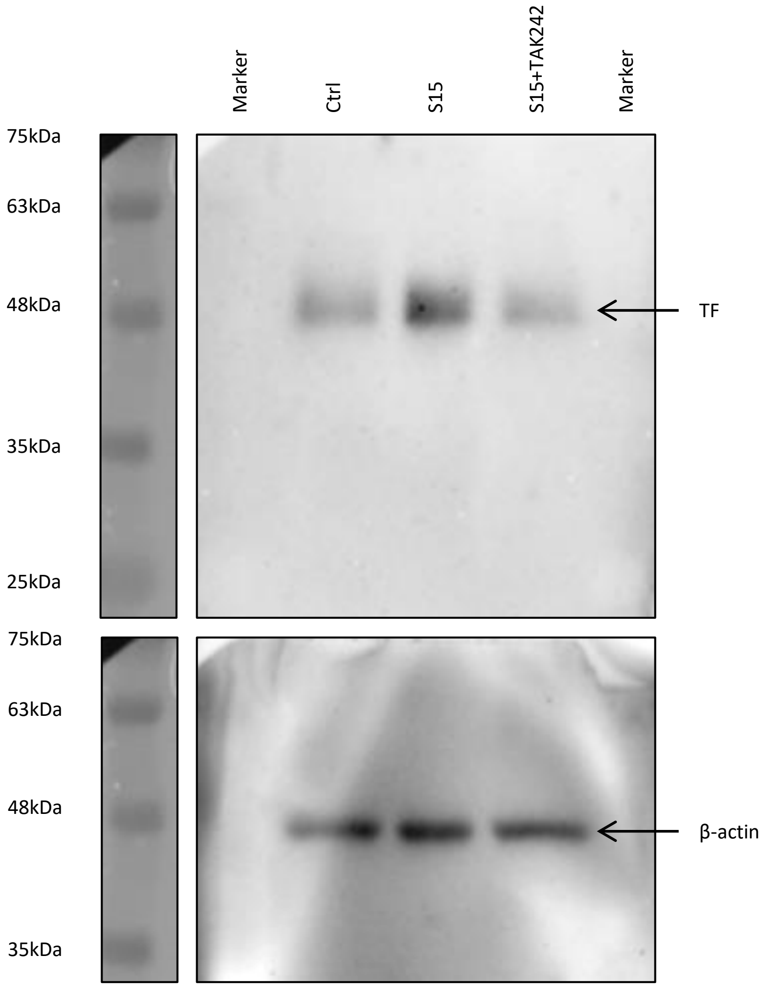

Supplement: Supplementary file 3 — Additional file 2. [file 12964_2023_1397_MOESM2_ESM.pdf]
